# Construct validation of HER-WELL: a bifactor exploratory structural equation modeling evaluation of an eight-domain wellness scale for women

**DOI:** 10.3389/fgwh.2026.1903478

**Published:** 2026-08-18

**Authors:** Heba Yaagoub AlNujaidi, Saja A. Al-Rayes, Arwa Alumran, Omar Y. Almukhadhib, Danya Y. AlNujaidi, Ibrahim A. Alhafid, Ghada F. Alyousif

**Affiliations:** 1 College of Public Health, Imam Abdulrahman Bin Faisal University, Dammam, Saudi Arabia; 2 Department of Obstetrics and Gynecology, Imam Abdulrahman Bin Faisal University, National Guard Health Affairs, Dammam, Saudi Arabia; 3 College of Medicine, Imam Abdulrahman Bin Faisal University, Dammam, Saudi Arabia; 4 Department of Family and Community Medicine, College of Medicine, Imam Abdulrahman Bin Faisal University, Dammam, Saudi Arabia

**Keywords:** Arab women, bifactor ESEM model, construct validity, cultural adaptation, factor structure, HER-WELL scale, holistic wellness

## Abstract

**Background:**

Wellness is a multidimensional latent construct whose valid assessment requires psychometrically robust, culturally adapted instruments; however, existing scales have been predominantly developed in Western contexts and remain inadequate for capturing the gendered and sociocultural dimensions of women's wellness in non-Western settings. This study aimed to develop and psychometrically validate the HER-WELL scale, a culturally adapted multidimensional measure of holistic wellness for Saudi women.

**Methods:**

Scale development followed Boateng et al.'s (2018) framework, and construct validity was tested in RStudio and Mplus by comparing six measurement models (final CFA, ESEM, second-order CFA, bifactor CFA, second-order ESEM, bifactor ESEM) estimated with robust WLSMV and TARGET rotation in a stratified community sample of Saudi women (N = 1,068; 531 employed, 537 non-employed), with model fit (CFI, TLI, RMSEA, SRMR) evaluated against conventional and Dynamic Fit Index cutoffs.

**Results:**

HER-WELL items showed adequate variability, with the highest endorsement for Home Environment, social, and spiritual wellness and the lowest for physical wellness. Across employed, non-employed, and full samples, bifactor ESEM consistently provided the best representation of the data, with excellent fit (CFI ≈.99, TLI ≈.98, RMSEA ≈.03, SRMR ≈.02) that exceeded both conventional and DFI-tailored cutoffs. This structure supported a strong general women's wellness factor, with Physical, Financial, Mental, and Occupational wellness retaining sufficient domain-specific variance, while Emotional, Social, Spiritual, and Home Environmental wellness were more strongly saturated by the general factor and are best interpreted as expressions of overall wellness. Internal consistency was acceptable for most domains (*ω* and *α* typically ≥.80), with Home Environmental wellness showing the weakest reliability and specific-factor indices of any domain (*ω* ≈.77; AVE =.070–.122), and should be interpreted only via the total score.

**Conclusion:**

ER-WELL demonstrates strong psychometric properties as a culturally validated measure of holistic wellness for Saudi women. The bifactor ESEM solution supports the simultaneous use of overall wellness scores alongside domain-specific profiles for Physical, Financial, Mental, and Occupational wellness for research, surveillance, and intervention evaluation in Saudi Arabia and similar Arab and Muslim-majority contexts.

## Introduction

Accurate and culturally valid measurement of wellness is essential for guiding health promotion policy, designing targeted interventions, and monitoring population wellbeing. Although several wellness instruments, including the Perceived Wellness Survey and the Five Factor Wellness Inventory, have undergone psychometric evaluation, they were developed and validated primarily in Western general-population contexts and provide limited evidence regarding their suitability for capturing the gendered and culturally specific dimensions of wellness experienced by Arab and Saudi women. Given that wellness is a latent construct that cannot be directly observed, its assessment requires psychometrically validated scales that demonstrate robust construct validity across culturally distinct populations.

The most widely used wellness instruments include Swarbrick's Wellness Inventory, which operationalizes the Eight Dimensions of Wellness model (1), TestWell Wellness Inventory (2), the Wellness Evaluation of Lifestyle (3), the Perceived Wellness Scale (4), Five Factor Wellness Inventory (5), Body-Mind-Spirit Wellness Behavior and Characteristic Inventory (6), the Wellness Beliefs Scale (7), the Holistic Wellness Assessment (8), and the Salutogenic Wellness Promotion Scale (9). These instruments were developed primarily in Western contexts and designed for general populations, with limited attention to gender-specific or Arab cultural considerations. To date, no validated wellness scale tailored to the socio-cultural realities of Arab or Saudi women, representing a significant gap in both the measurement and promotion of women's wellness in the region.

The present scale is theoretically grounded in the Eight Dimensions of Wellness framework, initially conceptualized by Swarbrick (10) and recently validated through CFA (1). This framework views wellness as a holistic, multidimensional construct encompassing physical, emotional, social, spiritual, occupational, intellectual, Environmental, and financial well-being. While the framework has been widely applied in health promotion and academic settings, its operationalization often reflects Western cultural norms and is not gender specific. To ensure contextual relevance, the current study builds on earlier qualitative research with Saudi women that explored their lived experiences, perceptions, and priorities regarding wellness. This formative phase identified culturally specific interpretations of each dimension. It revealed unique aspects such as the importance of family roles, religious practices, and the influence of the home Environment that informed the adaptation and refinement of the eight dimensions and their items for the Saudi context, which can later be applied to Arab women, as they share a similar culture. Prior to construct validation, the content validity of HER-WELL was rigorously established through a systematic expert panel evaluation (11). Item-level and scale-level content validity indices confirmed that the scale's items adequately represent the eight wellness domains and are culturally relevant to Saudi women's lived experiences, providing a sound evidential basis for proceeding to psychometric validation.

A central challenge in validating multidimensional wellness scales is selecting an analytic framework that accurately reflects the theoretical structure of the construct. Standard confirmatory factor analysis (CFA) imposes the assumption that items load exclusively on their designated factor, with all cross-loadings constrained to zero. While this approach enables rigorous hypothesis testing of a pre-specified structure, it is often overly restrictive for constructs such as wellness, where dimensions are theoretically and empirically interrelated, and items may meaningfully reflect more than one domain (12–14). When this zero-cross-loading assumption is violated, CFA can yield inflated factor correlations and distorted reliability estimates, misrepresenting the true dimensionality of the scale (15). Exploratory structural equation modeling (ESEM) addresses this limitation by permitting small but freely estimated cross-loadings, producing more realistic inter-factor correlations and more accurate reliability coefficients (16).

A further theoretical question concerns the hierarchical organisation of wellness domains. Second-order CFA and second-order ESEM models test whether the observed correlations among first-order wellness domains can be parsimoniously accounted for by a single higher-order general wellness factor that is, whether the eight domains are best understood as distinct but intercorrelated dimensions whose covariance is driven by an overarching wellness construct (17). While second-order models are theoretically appealing, they impose a proportionality constraint on factor loadings that may not hold in practice, and they do not permit the direct estimation of the unique variance contributed by each domain, independent of the general factor. Bifactor modeling directly addresses this question by partitioning variance into a general wellness factor and orthogonal domain-specific factors, thereby clarifying whether a total wellness score, individual domain scores, or both are psychometrically defensible (18, 19). Integrating bifactor modeling with ESEM, in what is known as bifactor-ESEM, simultaneously accounts for item cross-loadings and the presence of a general factor, and has been shown to outperform simpler CFA and ESEM solutions in the validation of complex health and well-being measures (20, 21). Guided by these theoretical and psychometric considerations, the present study employs a systematic structural model comparison approach encompassing CFA, ESEM, and bifactor variants to identify the most appropriate and defensible representation of women's wellness as operationalized by HER-WELL.

Furthermore, perceptions of and priorities for wellness may vary by employment status, as employment can influence occupational, financial, and social dimensions, as well as the balance between personal and professional life. Recent studies across the U.S., South Korea, the UK, and Australia reveal how employment and unemployment affect subjective well-being, perceived health, and mental state. These differences are often moderated by gender, socioeconomic status, and job security (22–25). Building on this evidence, the present study analyzed employed and non-employed Saudi women in parallel, comparing the measurement structure and quality indices of HER-WELL across these two groups to identify potential group-specific patterns that could inform targeted wellness interventions.

Accordingly, this study aims to develop and validate HER-WELL, an eight-domain women's wellness scale for Saudi women, using a comprehensive structural model comparison framework. All models will be tested separately in employed and non-employed women to examine whether the measurement structure and psychometric quality indices are comparable across employment status. The primary objective is to establish whether HER-WELL supports a psychometrically robust general wellness factor alongside interpretable domain scores, thereby providing a culturally grounded overall wellness index for Saudi women, complemented by informative domain-level profile scores.

## Methods

### Study design

This study adopted a cross-sectional survey design. Data were collected by the Sharik Association for Health Research using the ZdataCloud® platform, a research data acquisition framework that integrates eligibility screening, sampling control, and secure data storage to minimize anthropogenic sampling bias and ensure data integrity (26, 27).

### Participants, sampling, and sample size

A stratified quota sampling strategy was used to ensure representation across both age groups and employment status within the 13 administrative regions of Saudi Arabia. Representation was guided by prior research recommending the use of distinct life-stage categories in the population (28–30). Accordingly, the sample was stratified into three age groups: 18–29 years (young adulthood), 30–45 years (middle adulthood), and 46–65 years (late adulthood). Within each region, quotas were set for employed and non-employed women across age groups to approximate the national population structure. Participants were Saudi women aged 18–65 years, Arabic literate, and residing in Saudi Arabia. Respondents who did not meet the inclusion criteria were excluded from the study.

Sample size adequacy was evaluated using multiple criteria. The widely cited rule of thumb is to use a sample-to-item ratio of 5–10 participants per item (31). Given that the HER-WELL instrument initially comprised 54 items for both employed and non-employed women, the minimum recommended sample size was 270 participants (54 × 5), with a preferred target of 540 participants (54  ×  10). The achieved stratified national sample (N = 1,068; approximately 500 + women in each employment group) exceeded these thresholds and meets common recommendations for structural equation modeling and multi-group analyses

The literature further recognizes that sample size requirements in SEM depend not only on absolute sample size but also on data quality. Specifically, high communalities, low cross-loadings, and multiple strong item-factor loadings can support stable estimation with smaller samples (13, 32).. The sample of 1,068 Saudi women (531 employed; 537 non-employed) substantially exceeded all minimum thresholds and provided sufficient power for confirmatory factor analysis and structural equation modeling. Employment status was coded into two groups. The employed group included women working as employees or self-employed business owners. The non-employed group included students, homemakers, and retired women who were not in paid work and not actively seeking employment

### Participant characteristics

Participants were proportionally distributed across the 13 regions and three age groups within both employment strata. Table 1 presents the cross-tabulation of region, age group, and employment status, demonstrating broad coverage of the national female population.

**Table 1:** Participant characteristics across region, age, and employment status.

Region	18–29 yrs (Young adult)	30–45 yrs (Middle adult)	46–65 yrs (Late adulthood)	Total
	Employed/non-employed	Employed/non-employed	Employed/non-employed	
Riyadh	15/22	15/8	15/15	**90**
Qassim	10/18	18/15	13/12	**86**
Al-Baha	10/13	18/16	12/13	**82**
Madinah	13/14	15/16	14/14	**86**
Makkah	12/14	16/16	13/13	**84**
Eastern Province	15/16	17/16	15/16	**94**
Al-Jawf	12/13	17/14	12/13	**81**
Northern Borders	13/14	20/14	8/12	**81**
Tabuk	12/12	16/16	13/13	**82**
Hail	7/8	9/6	4/11	**45**
Jizan	12/14	20/15	18/13	**92**
Asir	13/13	15/17	12/13	**83**
Najran	12/13	16/15	14/12	**82**
**Total**	**156/183**	**212/184**	**163/170**	**1068**

Bold = total across all 13 administrative areas.

### Instrument HER-WELL scale

The HER-WELL (Holistic Empowerment and Resilience–Wellness) Scale was developed by the authors, drawing on the Eight Dimensions of Wellness framework (10), which was theoretically refined and culturally adapted through prior qualitative research with Saudi women. Prior to construct validation, the face and content validity of HER-WELL were rigorously established through a systematic expert panel evaluation (11). The scale begins with nine socioeconomic items adopted from published Saudi socioeconomic indices (33). The wellness scale comprises 54 items distributed across eight dimensions: physical, emotional, mental, social, spiritual, home Environment, financial, and occupational wellness. To address the contextual differences in women's roles, the occupational dimension included six items tailored to employed women and six alternative items for non-employed women; branching logic within the survey software ensured that participants responded only to items relevant to their employment status.

The medical literature indicates that Likert scales should at least have five response options to guarantee a reliable outcome (34). All items were rated on a 5-point Likert scale ranging from 1 (never) to 5 (always), with higher scores indicating greater wellness. A 5-point response format was selected based on evidence demonstrating its superior reliability, distributional properties, and respondent acceptability compared with scales with fewer response options (35, 36). Each dimension contained at least four items, consistent with recommended indicator thresholds for identifying stable constructs in SEM (37).

### Data collection procedure

Data collection was conducted between 26 February and 30 June 2025. Trained data collectors administered the survey by telephone using a standardized protocol that included eligibility screening, informed consent, and survey administration. Verbal informed consent was obtained from all participants prior to data collection. The ZdataCloud® system automatically directed each respondent to the appropriate occupational wellness item set (employed or non-employed) based on reported employment status, thereby preventing misclassification and supporting data integrity.

### Data preparation and preliminary screening

Data preparation followed standard psychometric procedures:

- Missing data and attention checks: Cases with >20% missing responses or failed attention checks were removed prior to analysis. No missing cases were identified; the data collectors dropped that participant who didn't complete the survey.
- Reverse coding: Negatively worded items were reverse-coded so that higher scores consistently reflected higher wellness. For example, items expressing guilt about neglecting Qur’an recitation (spiritual domain, Sp6) or emotional strain when supporting others (emotional domain, Em3) were recoded to align directionally with positively worded items.
- Outlier detection: Multivariate outliers were identified using the Mahalanobis distance [*χ*^2^(df = 54, *p* < 0.001) = 91.67]. CFA models estimated with and without 99 flagged cases showed comparable fit; all cases were retained to preserve power.
- Assumption checks: Descriptive statistics, the KMO statistic, and Bartlett’s test of sphericity were examined. Results supported the suitability of the data for factor analysis and SEM.
- Normality assessment. Multivariate normality was assessed separately for the employed, non-employed, and full samples using tests of multivariate skewness and kurtosis. All datasets showed substantial deviations from multivariate normality (skewness test statistics: 70,052.53, 73,185.14, and 75,020.75; excess kurtosis: 131.47, 157.43, and 232.62; all *p* < 0.001), justifying the use of robust estimators for ordinal data in subsequent CFA and SEM analyses.

### Statistical analysis

Construct validity was assessed within the SEM framework, specifically using confirmatory factor analysis (CFA), exploratory SEM (ESEM), and hierarchical models [Bifactor Models, Second-Order (Higher-Order)]. These second-generation techniques that extend traditional first-generation methods by simultaneously estimating latent factor structures, modeling measurement error, and enabling rigorous multi-model comparison (38)

### Estimator and software

Because all HER-WELL Scale items employ a five-point Likert response format, items were treated as ordered categorical (ordinal) variables rather than continuous indicators. The application of maximum likelihood (ML) estimators to ordinal data violates the assumption of multivariate normality, leading to biased standard errors, parameter estimates, and fit indices (39). Accordingly, all CFA and SEM models were estimated using the Weighted Least Squares Mean and Variance Adjusted (WLSMV) estimator, which models polychoric correlations and accounts for the discrete nature of the response scale (40). This decision was further supported by examination of multivariate normality, which confirmed a significant departure from normality in the data. Primary analyses were conducted in RStudio (2025.09.0 + 387) using the lavaan package (version 0.6-21; 41). Bifactor ESEM and second-order ESEM specifications, which exceed the current parameterization capabilities of lavaan, were estimated in Mplus (Version 9, 2025; 42). Model comparisons across all six structures were subsequently conducted using consistent fit-index criteria in both software Environments.

### Analytical sample stratification

The fact that the employed and non-employed occupational wellness items are mutually exclusive prevented their simultaneous use in a single analytical model without causing structural non-equivalence. Accordingly, three analytical samples were defined: (1) the employed subsample (*n* = 531), including the six occupational items for employed women; (2) the non-employed subsample (*n* = 537), including the six occupational items for non-employed women; and (3) the full sample (*n* = 1,068), with the occupational wellness dimension excluded entirely. This stratification strategy ensured that each model remained structurally coherent and prevented item-level non-equivalence across groups from contaminating estimates of model fit.

### Construct validity: model comparison strategy

To rigorously evaluate the dimensional structure of the HER-WELL Scale, a systematic model-comparison approach was adopted rather than relying on a single CFA model as shown in Table 2, consistent with recommendations in scale development research (43). The sequential progression from CFA through ESEM to higher-order and bifactor specifications was not predetermined but emerged through careful, iterative consideration of model fit evidence, theoretical alignment, and extensive review of the contemporary psychometric literature on factorial model selection in multidimensional health and psychological constructs (20, 37, 44, 45).

**Table 2:** Overview of the CFA baseline and Six measurement models for wellness scale evaluation.

Model	Description
CFA–Initial (Baseline model)	First-order independent clusters model (ICM-CFA); all cross-loadings constrained to zero
Phase 1: Confirmatory Factor Analysis (CFA)	
1. CFA – Final	Respecified ICM-CFA following systematic item refinement across all three samples
Phase 2: Exploratory Structural Equation Modeling (ESEM)	
2. ESEM	Exploratory SEM with target rotation; cross-loadings freely estimated
Phase 3: Higher-Order & Bifactor Models	
3. Second-Order CFA	Wellness modelled as a second-order latent factor over eight first-order dimensions
4. Bifactor CFA	General wellness factor with uncorrelated orthogonal specific factors (ICM-CFA parameterisation)
5. Second-Order ESEM	Second-order structure estimated with ESEM cross-loadings
6. Bifactor ESEM	General wellness factor with ESEM-specified specific factors; estimated in Mplus

*Note.* ICM-CFA=Independent Cluster Model CFA; ESEM=Exploratory Structural Equation Modeling. All models were generated using RStudio and Mplus. CFA–Initial (baseline model) was estimated as a diagnostic independent-clusters CFA and was not included among the six competing measurement models.

Item refinement in the CFA-Final model was guided by cross-group consistency: items were retained for deletion only when low or problematic factor loadings were observed consistently across all three analytical samples (employed, non-employed, and full sample), ensuring that model modifications were not sample-specific artifacts. The primary ESEM model employed target rotation, given the *a priori* assignment of items to the eight wellness dimensions, consistent with recommendations to use target rotation when substantive measurement theory is available. A Geomin-rotated solution was additionally estimated to verify that the improved fit was not rotation-dependent; both solutions yielded virtually identical global fit indices, confirming the robustness of the ESEM results. Because WLSMV does not provide AIC or BIC and its chi-square cannot be used for standard difference testing, nested model comparisons were conducted using the DIFFTEST robust chi-square procedure, supplemented by evaluation of changes in CFI, TLI, RMSEA, and SRMR across competing models (46). Model selection was guided by three criteria considered jointly: (a) statistical fit relative to fixed cutoff thresholds, (b) theoretical interpretability of factor loadings and cross-loadings, and (c) parsimony relative to model complexity (44). For the bifactor model, four complementary indices were examined to evaluate the strength and reliability of the general wellness factor: (a) Omega total (*ω*), reflecting the overall reliability of the composite score; (b) Omega hierarchical (*ω*ₕ), indicating the proportion of reliable total score variance attributable to the general factor; (c) Explained Common Variance (ECV), reflecting the proportion of common variance accounted for by the general factor relative to all factors combined; and (d) the H index and Factor Determinacy (FD), which jointly assess the replicability and precision of the general factor across samples (19, 20). These indices were computed for the general wellness factor (G) to determine whether the HER-WELL total score primarily reflects a single dominant wellness dimension or a genuinely multidimensional profile.

### Model Fit evaluation and tailored cutoff criteria

Model fit was assessed using the Comparative Fit Index (CFI), Tucker–Lewis Index (TLI), Root Mean Square Error of Approximation (RMSEA), and the Standardized Root Mean Square Residual (SRMR). Conventional fixed cutoff values (e.g., Hu & Bentler, 1999: CFI ≥.95, RMSEA ≤.06) were not applied in isolation (47). Methodological literature has demonstrated that these universal thresholds generalize poorly across models that differ in factor structure, number of items, loading strength, and estimator type, and that their application to WLSMV-estimated ordinal models is particularly problematic (48, 49).

Several tailored cutoff methods have been proposed as alternatives to fixed thresholds. The *χ*^2^ distribution-based approach uses parametric distributions to derive RMSEA cutoffs and controls Type I/II errors using tools such as semPower (50). Regression-based methods apply meta-regressions from simulations (51), prioritizing speed over broad coverage. thereby maximizing flexibility across indices (52). Bootstrap procedures resample the data nonparametrically to obtain error-balanced cutoffs, as implemented in lavaan/simsem (53). The simulation-cum-ROC approach combines Monte Carlo simulations with ROC analysis to derive optimal thresholds by maximizing Youden's index (54). Finally, Dynamic simulations employ Monte Carlo methods that match empirical conditions (e.g., ezCutoffs, Dynamic Fit Index),

The dynamic fit index (DFI) approach by McNeish, (2023a) was selected for the present study for two reasons (55). First, unlike the simulation-cum-ROC approach, which has been developed and validated primarily for simple CFA structures under maximum likelihood estimation, DFI is explicitly designed to generate model-specific cutoffs under the exact estimator, factor structure, number of indicators, and response distributions of the target model. Second, DFI directly accommodates the complexity of bifactor and ESEM specifications under WLSMV estimation with ordinal Likert items the precise conditions of the present analyses. Tailored cutoffs were therefore derived from a Monte Carlo simulation of the theory-driven measurement model. Table 3 presents the fixed and tailored fit index thresholds used for model evaluation.

**Table 3:** Fixed and tailored fit index thresholds used for model evaluation.

Model		CMIN	DF	P	CMIN/DF	TLI	CFI	RMSEA	SRMR
Fixed Cutoff Scores	(47)	—	—	>.05	≤ 3.00	≥.90	≥.95	Good fit <.08 Moderate fit.08 to.10 Poor >.1	≤.08
	(37) (N > 250 and variable >30)	—	—	Expected Sig *P*-value	≤ 3.00	≥.90	≥.90	<.07	≤.08
Tailored Cutoffs (Monte Carlo) Bifactor ESEM	Employed	—	—	—	—	≥ 0.90	≥ 0.91	≤ 0.052	≤ 0.09
	Non-employed	—	—	—	—	≥ 0.95	≥ 0.94	≤ 0.041	≤ 0.073
	Full Sample	—	—	—	—	≥ 0.93	≥ 0.92	≤ 0.052	≤ 0.08

### Measurement quality criteria

Given that the model comparison yielded both ESEM and bifactor ESEM as candidate solutions, measurement quality was evaluated using two complementary sets of criteria reflecting the distinct properties of each model class. For the ESEM solutions, standard reflective measurement criteria were applied: indicator loadings, internal consistency via composite reliability (*ρ*_C) and McDonald's *ω*, convergent validity via average variance extracted (AVE), and discriminant validity via the heterotrait–monotrait ratio (HTMT) (56). It is noted that *ω* values in ESEM are typically lower than in ICM-CFA due to the presence of cross-loadings, which is statistically expected and does not indicate poor reliability (44). For the bifactor solution, standard CFA criteria are insufficient because items simultaneously load on a general factor and specific factors; accordingly, bifactor-specific indices — omega hierarchical (*ω*ₕ), omega total (*ω*), explained common variance (ECV), the H index, and factor determinacy (FD) — were used to evaluate the strength, dominance, and replicability of the general wellness factor (19, 57). Table 4 presents the full set of metrics and recommended thresholds applied across both model types.

**Table 4:** Model measurement quality criteria and thresholds.

Measurement	Criterion	Key Metrics	Recommended Thresholds	Reference
Model Measurement (CFA/ESEM)	Indicator Loadings	Standardized *λ* (target loadings); cross-loadings (ESEM)	Target *λ* ≥ 0.50 acceptable; ≥ 0.708 strict; cross-loadings <.20 (small) to < 0.30 (acceptable)	(58)
	Internal Consistency	Composite reliability (*ρ*C); McDonald's *ω*	*ρ*C/*ω* ≥ 0.70 (≥ 0.60 exploratory); ≤ 0.95; note: ω values tend to be lower in ESEM than CFA, which is expected and acceptable	
	Convergent Validity	AVE	≥ 0.50 (acceptable <0.50 if ρ_C/*α* > 0.70)	
	Discriminant Validity	HTMT (preferred) more than Fornell–Larcker	< 0.85 (conservative) or < 0.90 (lenient); in ESEM, lower factor correlations provide additional evidence	
Bifactior Model	General Factor Strength	Omega hierarchical (ωₕ)	≥.70–.80 indicates general factor accounts for most reliable variance	(19)
	Total Score Reliability	Omega total (ω)	≥.70 acceptable; value reflects total reliable variance across general and specific factors	
	General Factor Dominance	Explained Common Variance (ECV)	≥.50 suggests general factor dominance; ≥.70–.80 supports essentially unidimensional scoring.	
	Factor Replicability	H index	≥.70 acceptable; ≥.80 good; indicates stability of the general factor across different samples.	
	Factor Precision	Factor Determinacy (FD)	≥.90 indicates high precision in estimating the general factor	

## Result

### Overview of analytic steps

As outlined in the Methods, model evaluation proceeded in two phases. First, six competing measurement models were estimated to identify the most appropriate structural representation of the HER-WELL construct: CFA–Final, ESEM, second-order CFA, bifactor CFA, second-order ESEM, and bifactor ESEM. All models were estimated with WLSMV for ordered categorical items, and global fit was assessed using CFI, TLI, RMSEA, and SRMR, with model-specific Dynamic Fit Index (DFI) cutoffs derived from Monte Carlo simulations. Second, the best-fitting solutions were subjected to detailed measurement evaluation, including indicator loadings, internal consistency, and convergent and discriminant validity for the CFA/ESEM models, as well as bifactor-specific indices (*ω*, ω_h, ECV, H, FD) for the bifactor ESEM solution. All analyses were conducted separately for employed women, non-employed women, and the full sample, allowing assessment of the stability of the measurement structure across employment groups.

### Descriptive statistics and normality

Descriptive statistics for all 54 HER-WELL scale items were computed separately for the employed sample (*n* = 531), the non-employed sample (*n* = 537), and the full combined sample (*n* = 1,068), and are presented in **Supplementary Appendix 1**. Item-level means across the three samples ranged from 2.91 to 4.82. Standard deviations ranged from 0.56 to 1.45, reflecting moderate-to-high within-sample variability at the item level, consistent with expectations for a multidimensional wellness scale administered to a heterogeneous community sample. No floor effects were observed at the item or domain level. However, inspection of the upper end of the distribution revealed a pronounced ceiling effect concentrated in the Spiritual Wellness domain, discussed below.

At the domain level, Home Environment Wellness yielded the highest average item means in the Employed and Full samples (Employed: M = 4.29, SD = 0.99; Full sample: M = 4.23, SD = 1.04), followed closely by Social Wellness (Employed: M = 4.26, SD = 0.94; Full sample: M = 4.21, SD = 0.99). In the Non-employed sample, Spiritual Wellness yielded the highest average item mean (M = 4.24, SD = 0.71), followed by Social Wellness (M = 4.22, SD = 0.96) and Home Environment Wellness (M = 4.21, SD = 1.00). Spiritual Wellness means were similar across all three samples (Employed: M = 4.19, SD = 0.74; Non-employed: M = 4.24, SD = 0.71; Full sample: M = 4.17, SD = 0.80). Physical Wellness yielded the lowest average item means across all samples (Employed: M = 3.71, SD = 1.16; Non-employed: M = 3.58, SD = 1.19; Full sample: M = 3.63, SD = 1.19). Domain-level means were broadly consistent across employed and non-employed subsamples, suggesting comparability of wellness endorsement patterns across employment groups. Notably, item means for Sp2–Sp5 ranged from 4.67 to 4.82, with the 25th percentile equalling the scale maximum (5) in all three samples indicating that at least 75% of respondents selected the highest response category on these items, consistent with severe negative skew (−2.57 to −4.03) and kurtosis (up to 17.1).

Inspection of item-level skewness and kurtosis indices revealed that most items exhibited negative skewness, indicating a tendency for responses to cluster toward higher endorsement values, a pattern commonly observed in wellness and positive health scales administered to community samples. In the employed sample, skewness values ranged from −2.73 to −0.09 (*M* = −1.13), with four Spiritual Wellness items (Sp2, Sp3, Sp4, Sp5) exceeding the |2.0| threshold for severe skewness, and 25 items falling within the moderate range (1.0 < |Skew| ≤ 2.0). In the non-employed sample, skewness ranged from −4.03 to 0.18 (*M* = −1.10), with five Spiritual Wellness items (Sp1–Sp5) showing severe skewness. The full sample yielded a comparable pattern, with five severely skewed items and 19 items in the moderate range. With respect to kurtosis, the majority of items fell within acceptable bounds; however, three to four Spiritual Wellness items (Sp2–Sp5) exhibited severe kurtosis (|*K*| > 7) across all three samples, with a further two to four items showing moderate kurtosis (3 < |*K*| ≤ 7). Taken together, the elevated means, severe negative skew, and high kurtosis values for Sp2–Sp5 constitute a severe ceiling effect in the spiritual Wellness domain: responses were heavily concentrated in the top response category, compressing variance and limiting the domain's capacity to discriminate among respondents at the high end of the construct. This pattern is consistent with the highly positively valenced wording of the Spiritual Wellness items, which elicit near-universal endorsement among Saudi women, for whom faith and religious practice represent central and normative dimensions of wellbeing. These distributional findings confirm the appropriateness of WLSMV estimation, which accommodates non-normal ordinal response data without requiring multivariate normality assumptions.

### CFA–initial (baseline model) and item refinement

Inspection of standardized factor loadings, factor-level reliability, and AVE across employed, non-employed, and full-sample CFAs indicated that a small number of items were not functioning adequately. Specifically, three physical wellness items (PH1, PH3, PH4) had consistently weak loadings (around.40–.60) and suppressed the AVE of the physical factor below the recommended.50 threshold across all three samples, indicating limited shared variance with the intended construct. In addition, one emotional wellness item (Em3) and one spiritual wellness item (Sp6) showed negative weak standardized loadings in each sample, suggesting that these reverse-coded items were not operating as intended and were psychometrically inconsistent with the remaining indicators. Given the cross-sample consistency of these problems and to improve convergent validity and interpretability, PH1, PH3, PH4, Em3, and Sp6 were removed from the measurement model. The refined 49-item measurement model retained the following item counts per domain, consistent across all three subsamples: Physical Wellness (7 items), Emotional Wellness (8 items), Environmental Wellness (4 items), Social Wellness (7 items), Spiritual Wellness (6 items), Financial Wellness (5 items), Mental Wellness (6 items), and Occupational Wellness (6 items). All subsequent models (CFA–Final, ESEM, second-order, and bifactor models) were therefore estimated using the refined 49-item set.

### Global model Fit comparison

Global fit indices for the six competing measurement models across the three analytical samples are reported in Table 5. For all samples, the CFA models (final) and the second-order CFA models achieved acceptable but clearly suboptimal fit, with CFI and TLI values generally below the conventional.95 benchmark and RMSEA values in the.055–.063 range, indicating only adequate approximation of the observed covariance structure. Allowing cross-loadings through ESEM substantially improved fit relative to the CFA solutions, yielding higher CFI/TLI values and lower RMSEA/SRMR, but these models still did not consistently show the strongest fit across all indices and samples.

**Table 5:** Global model Fit comparison for a CFA baseline and Six competing measurement models across three analytical samples.

Sample	Model	*χ*^2^ (df, p) Scaled	CFI Scaled	TLI Scaled	RMSEA Scaled (90% CI)	SRMR Scaled	Meets Fixed Cutoffs?
Employed	CFA–Initial (Baseline model)	3087.665 (1099, *p* < 0.001)	0.924	0.922	0.058 [0.056–0.061]	0.076	—
	CFA–Final	3486.053 (1349, *p* < 0.001)	0.927	0.920	0.055 [0.052–0.057]	0.076	—
	ESEM	1284.750 (812, *p* < 0.001)	0.983	0.975	0.033 [0.030–0.037]	0.029	**✓**
	Second-Order CFA	3181.241 (1119, *p* < 0.001)	0.924	0.921	0.059 [0.057–0.061]	0.083	—
	Bifactor CFA	2641.566 (1078, *p* < 0.001)	0.943	0.937	0.052 [0.050–0.055]	0.072	—
	Second-Order ESEM	2601.785 (1086, *p* < 0.001)	0.944	0.940	0.051 [0.049–0.054]	0.072	—
	Bifactor ESEM	1143.330 (771, *p* < 0.001)	0.985	0.977	0.030 [0.026–0.034]	0.024	✓
Non-employed	CFA–Initial (Baseline model)	2382.924 (1099, *p* < 0.001)	0.957	0.954	0.047 [0.044–0.049]	0.056	**✓**
	CFA–Final	2815.848 (1349, *p* < 0.001)	0.953	0.951	0.045 [0.043–0.047]	0.058	**✓**
	ESEM	1244.530 (812, *p* < 0.001)	0.986	0.979	0.032 [0.028–0.035]	0.028	**✓**
	Second-Order CFA	2656.663 (1119, *p* < 0.001)	0.949	0.946	0.051 [0.048–0.053]	0.063	—
	Bifactor CFA	2310.273 (1078, *p* < 0.001)	0.959	0.955	0.046 [0.044–0.049]	0.056	**✓**
	Second-Order ESEM	2223.858 (1088, *p* < 0.001)	0.962	0.959	0.044 [0.042–0.047]	0.055	**✓**
	Bifactor ESEM	1110.267 (771, *p* < 0.001)	0.988	0.981	0.029 [0.025–0.032]	0.024	✓
Full sample	CFA–Initial (Baseline model)	4594.917 (1059, *p* < 0.001)	0.935	0.930	0.056 [0.054–0.058]	0.062	—
	CFA–Final	3872.147 (839, *p* < 0.001)	0.941	0.937	0.058 [0.056–0.060]	0.061	—
	ESEM	1523.290 (623, *p* < 0.001)	0.983	0.975	0.037 [0.034–0.039]	0.025	**✓**
	Second-Order CFA	4425.656 (853, *p* < 0.001)	0.931	0.927	0.063 [0.061–0.064]	0.069	—
	Bifactor CFA	3756.466 (817, *p* < 0.001)	0.943	0.937	0.058 [0.056–0.060]	0.060	—
	Second-Order ESEM	3188.276 (815, <.001)	0.954	0.949	0.052 [0.050–0.054]	0.056	**✓**
	Bifactor ESEM	1236.664 (587, *p* < 0.001)	0.986	0.978	0.032 [0.030–0.035]	0.020	✓
Conventional Cutoff Thresholds	***Recommended threshold***	***p*** ***<*** 0***.05 (df-sensitive)***	***≥.950***	***≥.950***	***≤.060***	***≤.080***	**✓**
Tailored cutoffs in bifactor ESEM	***Employed***	***-***	***≥ 0.91***	***≥ 0.90***	***≤ 0.052–0.053***	***≤ 0.09–0.10***	**✓**
	***Non-employed***	***-***	***≥ 0.95***	***≥ 0.94***	***≤ 0.041***	***≤ 0.073***	**✓**

χ2 = chi-square goodness-of-fit statistic; df, degrees of freedom; CFI, Comparative Fit Index; TLI, Tucker–Lewis Index; RMSEA, Root Mean Square Error of Approximation; SRMR, Standardised Root Mean Square Residual. All models were estimated using the robust WLSMV estimator treating items as ordered categorical. Fit indices are scaled (robust) values. Bold rows indicate the best-fitting model (Bifactor ESEM) in each sample. ✓ = all four conventional cutoffs met simultaneously (CFI/TLI ≥.95; RMSEA ≤.06; SRMR ≤.08). Full Sample excludes Occupational Wellness items. Conventional cutoffs from Hu & Bentler (1999); DFI-tailored cutoffs (McNeish, 2023).

**Figure 1:**
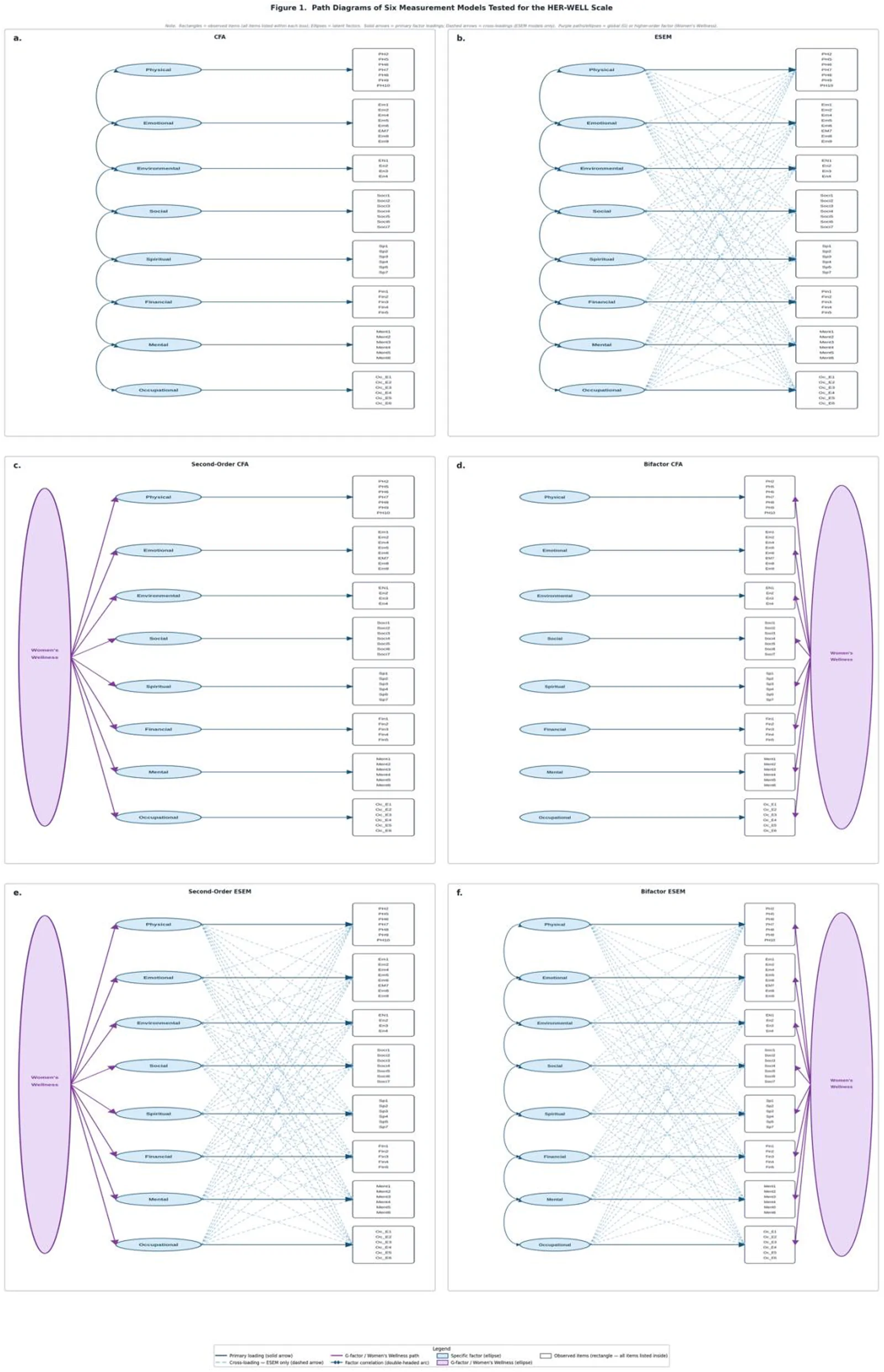
Path diagrams of six measurement models tested for the HER-WELL scale.

Across analyses of the employed, non-employed, and full samples, models that combined a general factor with specific domain factors systematically outperformed both the correlated-factor CFA and the higher-order models. In particular, the bifactor ESEM specification provided the best overall fit in all three samples, with CFI values between 0.985 and 0.988, TLI values between 0.977 and 0.981, RMSEA values between 0.029 and 0.032, and SRMR values between 0.020 and 0.024, each comfortably meeting or exceeding conventional cutoff criteria. Bifactor CFA and second-order ESEM also improved upon the pure CFA models but did not achieve the same level of fit as the bifactor ESEM, particularly in RMSEA and SRMR. Taken together, these results indicate that a model in which items load simultaneously on a general women's wellness factor and on their specific domain factors (bifactor ESEM) provides the most adequate and parsimonious representation of the HER-WELL measurement structure across employment groups. Figure 1 presents the path diagrams of the six competing measurement models evaluated in the present study, ranging from the CFA to the final Bifactor ESEM solution.

### Model selection and DFI validation

**Figure 2:**
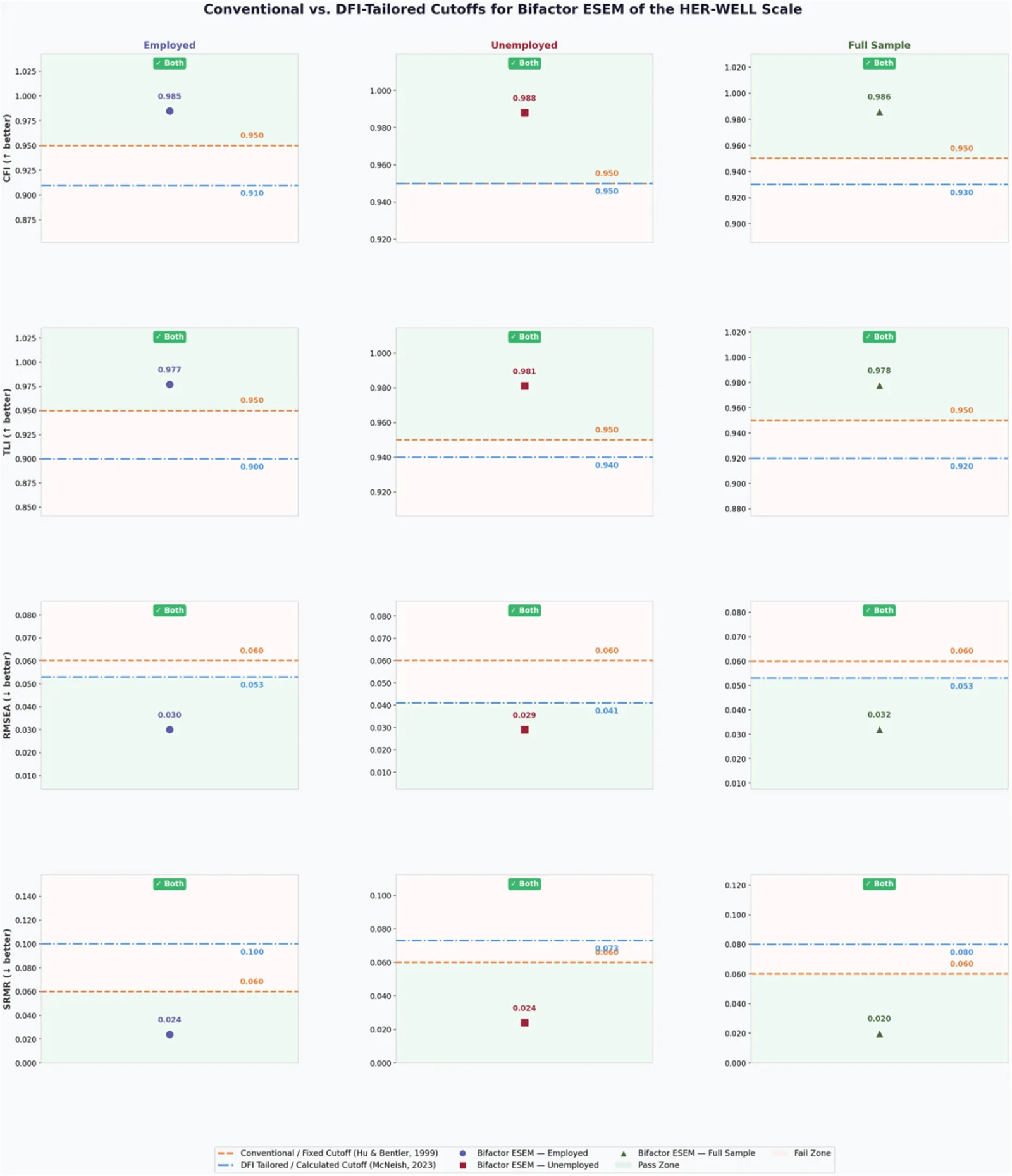
Comparison of conventional fixed cutoffs vs. dynamic fit index (DFI)- tailored cutoffs in a bifactor ESEM model.

To formally select the final measurement structure, the Bifactor ESEM model was evaluated against both conventional fixed fit-index cutoffs and Monte Carlo–based Dynamic Fit Index (DFI) tailored cutoffs for each sample. Figure 2 presents a comparison of conventional fixed cutoffs vs. dynamic fit index (DFI)- tailored cutoffs in the bifactor ESEM model. In the employed group, the Bifactor ESEM showed excellent fit (CFI=0.985, TLI=0.977, RMSEA=0.030, SRMR=0.024), comfortably exceeding conventional criteria (CFI/TLI ≥ 0.95, RMSEA ≤ 0.06, SRMR ≤ 0.08) and also surpassing the DFI thresholds derived under the correctly specified model (CFI ≥ 0.91, TLI ≥ 0.90, RMSEA ≤ 0.053, SRMR ≤ 0.10). The same pattern emerged in the non-employed sample, where the Bifactor ESEM again exhibited very strong fit (CFI=0.988, TLI=0.981, RMSEA=0.029, SRMR=0.024) and met or exceeded the more stringent DFI cutoffs (CFI ≥ 0.95, TLI ≥ 0.94, RMSEA ≤ 0.041, SRMR ≤ 0.073).

In the full combined sample, the Bifactor ESEM yielded similarly favorable indices (CFI=0.986, TLI=0.978, RMSEA=0.032, SRMR=0.020), again outperforming conventional benchmarks and lying well within the DFI-tailored bounds (CFI ≥ 0.93, TLI ≥ 0.92, RMSEA ≤ 0.053, SRMR ≤ 0.08). Overall, bifactor ESEM was retained as the preferred representation of HER-WELL because it combined superior global fit with theoretically coherent cross-loadings and a substantively meaningful decomposition of variance into a general wellness factor and domain-specific factors. Simpler CFA-based models were rejected as inadequate, as their stronger constraints yielded poorer fit and a less realistic portrayal of the interrelated wellness dimensions, and were therefore not considered parsimonious in a substantive sense.

Figure 2 graphically compares, for each sample and each index (CFI, TLI, RMSEA, SRMR), the observed Bifactor ESEM values against both the traditional fixed cutoffs and the DFI thresholds, highlighting a consistent pattern: in all three groups, observed fit indices not only satisfy conventional standards but also provide a comfortable margin relative to the empirically derived DFI cutoffs. Taken together with the global model comparison, these results indicate that the Bifactor ESEM representation of HER-WELL offers an excellent approximation to the data-generating process across employment contexts, justifying its use as the final measurement model for subsequent reliability and validity analyses.

Figure 1 presents the path diagrams of the six competing measurement models evaluated in the present study, ranging from the CFA to the final Bifactor ESEM solution.

### Factor loadings and cross-loadings

#### ESEM factor loadings and cross-loadings

Table 6 presents the standardized primary factor loadings (*λ*) and associated standard errors derived from the ESEM solution across the employed, non-employed, and full samples. All loadings were estimated using WLSMV with THETA parameterization and orthogonal TARGET rotation. Consistent with ESEM conventions, primary loadings ≥.40 were regarded as meaningful and cross-loadings <.30 as negligible; the complete cross-loading matrices for all three samples are reported in Supplementary Appendices 2–4.

**Table 6:** Standardized factor loadings (λ) and standard errors from ESEM with WLSMV estimation across three groups.

Dimensions	Items	All women		Employed		Non-Employed	
		λ	SE	λ	SE	λ	SE
Physical	PH2	*0.250*ᶜ	0.036	—	—	*0.194*ᶜ	0.049
	PH5	*0.224*ᶜ	0.050	*0.236*ᶜ	0.067	*0.240*ᶜ	0.068
	PH6	0.330ᶜ	0.037	0.390ᶜ	0.046	*0.293*ᶜ	0.056
	PH7	0.831ᶜ	0.023	0.864ᶜ	0.030	0.786ᶜ	0.035
	PH8	0.805ᶜ	0.022	0.863ᶜ	0.029	0.776ᶜ	0.034
	PH9	0.812ᶜ	0.023	0.764ᶜ	0.027	0.815ᶜ	0.034
	PH10	0.766ᶜ	0.027	0.694ᶜ	0.031	0.809ᶜ	0.040
	*Mean λ*	*0.574*		*0.635*		*0.559*	
	*Range λ*	*0.224–0.831*		*0.236–0.864*		*0.194–0.815*	
Emotional	Em1	0.532ᶜ	0.055	0.626ᶜ	0.048	0.308ᶜ	0.060
	Em2	0.492ᶜ	0.047	0.518ᶜ	0.048	0.345ᶜ	0.063
	Em4	0.373ᶜ	0.039	0.542ᶜ	0.052	0.395ᶜ	0.057
	Em5	0.467ᶜ	0.047	—	—	0.643ᶜ	0.054
	Em6	0.511ᶜ	0.037	0.632ᶜ	0.042	0.507ᶜ	0.049
	Em7	0.535ᶜ	0.074	0.579ᶜ	0.057	0.607ᶜ	0.048
	Em8	0.540ᶜ	0.057	0.537ᶜ	0.046	0.718ᶜ	0.048
	Em9	0.556ᶜ	0.062	0.530ᶜ	0.045	0.571ᶜ	0.050
	*Mean λ*	*0.501*		*0.566*		*0.512*	
	*Range λ*	*0.373–0.556*		*0.518–0.632*		*0.308–0.718*	
Home environmental	En1	0.304ᶜ	0.066	—	—	0.389ᶜ	0.046
	En2	*0.271*ᶜ	0.055	—	—	0.321ᶜ	0.043
	En3	*0.274*ᶜ	0.072	0.436ᶜ	0.052	*0.209*ᶜ	0.061
	En4	*0.201*ᵇ	0.073	—	—	0.352ᶜ	0.052
	*Mean λ*	*0.262*		*0.436*		*0.318*	
	*Range λ*	*0.201–0.304*		*0.436–0.436*		*0.209–0.389*	
Social	Soci1	0.428ᶜ	0.052	—	—	*0.266*ᶜ	0.074
	Soci2	0.652ᶜ	0.049	0.657ᶜ	0.059	0.510ᶜ	0.060
	Soci3	0.833ᶜ	0.046	0.808ᶜ	0.054	0.650ᶜ	0.059
	Soci4	0.850ᶜ	0.042	0.815ᶜ	0.048	0.714ᶜ	0.056
	Soci5	0.375ᶜ	0.044	—	—	0.340ᶜ	0.055
	Soci6	0.504ᶜ	0.047	—	—	0.554ᶜ	0.057
	Soci7	0.433ᶜ	0.044	—	—	0.523ᶜ	0.049
	*Mean λ*	*0.582*		*0.760*		*0.508*	
	*Range λ*	*0.375–0.850*		*0.657–0.815*		*0.266–0.714*	
Spiritual	Sp1	0.778ᶜ	0.039	0.725ᶜ	0.057	0.696ᶜ	0.043
	Sp2	0.874ᶜ	0.028	0.890ᶜ	0.043	0.744ᶜ	0.038
	Sp3	0.817ᶜ	0.031	0.761ᶜ	0.049	0.788ᶜ	0.042
	Sp4	0.855ᶜ	0.032	0.860ᶜ	0.044	0.737ᶜ	0.043
	Sp5	0.692ᶜ	0.037	0.663ᶜ	0.051	0.627ᶜ	0.045
	Sp7	0.464ᶜ	0.044	—	—	0.411ᶜ	0.053
	*Mean λ*	*0.747*		*0.780*		*0.667*	
	*Range λ*	*0.464–0.874*		*0.663–0.890*		*0.411–0.788*	
Financial	Fin1	0.722ᶜ	0.031	0.737ᶜ	0.046	0.790ᶜ	0.042
	Fin2	0.764ᶜ	0.031	0.720ᶜ	0.047	0.900ᶜ	0.042
	Fin3	0.770ᶜ	0.029	0.744ᶜ	0.042	0.834ᶜ	0.041
	Fin4	0.619ᶜ	0.030	0.598ᶜ	0.045	0.665ᶜ	0.045
	Fin5	0.434ᶜ	0.035	0.407ᶜ	0.046	0.450ᶜ	0.051
	*Mean λ*	*0.662*		*0.641*		*0.728*	
	*Range λ*	*0.434–0.770*		*0.407–0.744*		*0.450–0.900*	
Mental	Ment1	0.670ᶜ	0.025	0.613ᶜ	0.035	0.610ᶜ	0.034
	Ment2	0.544ᶜ	0.029	0.554ᶜ	0.038	0.419ᶜ	0.042
	Ment3	0.623ᶜ	0.024	0.587ᶜ	0.037	0.589ᶜ	0.037
	Ment4	0.552ᶜ	0.033	0.551ᶜ	0.043	0.527ᶜ	0.038
	Ment5	0.634ᶜ	0.029	0.616ᶜ	0.039	0.654ᶜ	0.035
	Ment6	0.553ᶜ	0.029	0.527ᶜ	0.038	0.509ᶜ	0.040
	*Mean λ*	*0.596*		*0.575*		*0.551*	
	*Range λ*	*0.544–0.670*		*0.527–0.616*		*0.419–0.654*	
Occupational	Occup 1	—	—	0.527ᶜ	0.045	0.610ᶜ	0.049
	Occup 2	—	—	0.929ᶜ	0.021	0.645ᶜ	0.051
	Occup 3	—	—	0.961ᶜ	0.021	0.700ᶜ	0.052
	Occup 4	—	—	0.811ᶜ	0.032	0.812ᶜ	0.060
	Occup 5	—	—	0.813ᶜ	0.027	0.817ᶜ	0.050
	Occup 6	—	—	0.614ᶜ	0.044	0.519ᶜ	0.049
	*Mean λ*	*—*		*0.776*		*0.684*	
	*Range λ*	*—*		*0.527–0.961*		*0.519–0.817*	

ESEM, exploratory structural equation modeling. λ = standardized factor loading under WLSMV estimation, Theta parameterization, orthogonal target rotation. ᵃ*p* < 0.05. ᵇ*p* < 0.01. ᶜ*p* < 0.001. — = not estimated.

Primary loadings were statistically significant (*p* < 0.001) across all samples and domains. The Spiritual dimension yielded the strongest loadings (Full: *λ* = 0.464–.874; Employed: *λ* = 0.663–.890; Non-Employed: *λ* = 0.411–.788), followed by Financial (Full: *λ* = 0.434–.770), Social (Full: *λ* = 0.375–.850), and Physical (Full: *λ* = 0.224–.831). The Mental and Emotional domains showed moderate primary loadings (Mental Full: *λ* = 0.544–.670; Emotional Full: *λ* = 0.373–.556), while the Home Environmental domain produced the most modest estimates across all groups (Full: *λ* = 0.201–.304). The Occupational factor was estimable in the employed and non-employed samples only, yielding strong primary loadings in both groups (Employed: *λ* = 0.527–.961; Non-Employed: *λ* = 0.519–.817). Cross-loadings were generally small in magnitude, with the majority falling below.30, supporting the empirical distinctiveness of the eight wellness domains.

#### Bifactor ESEM factor loadings

**Figure 3:**
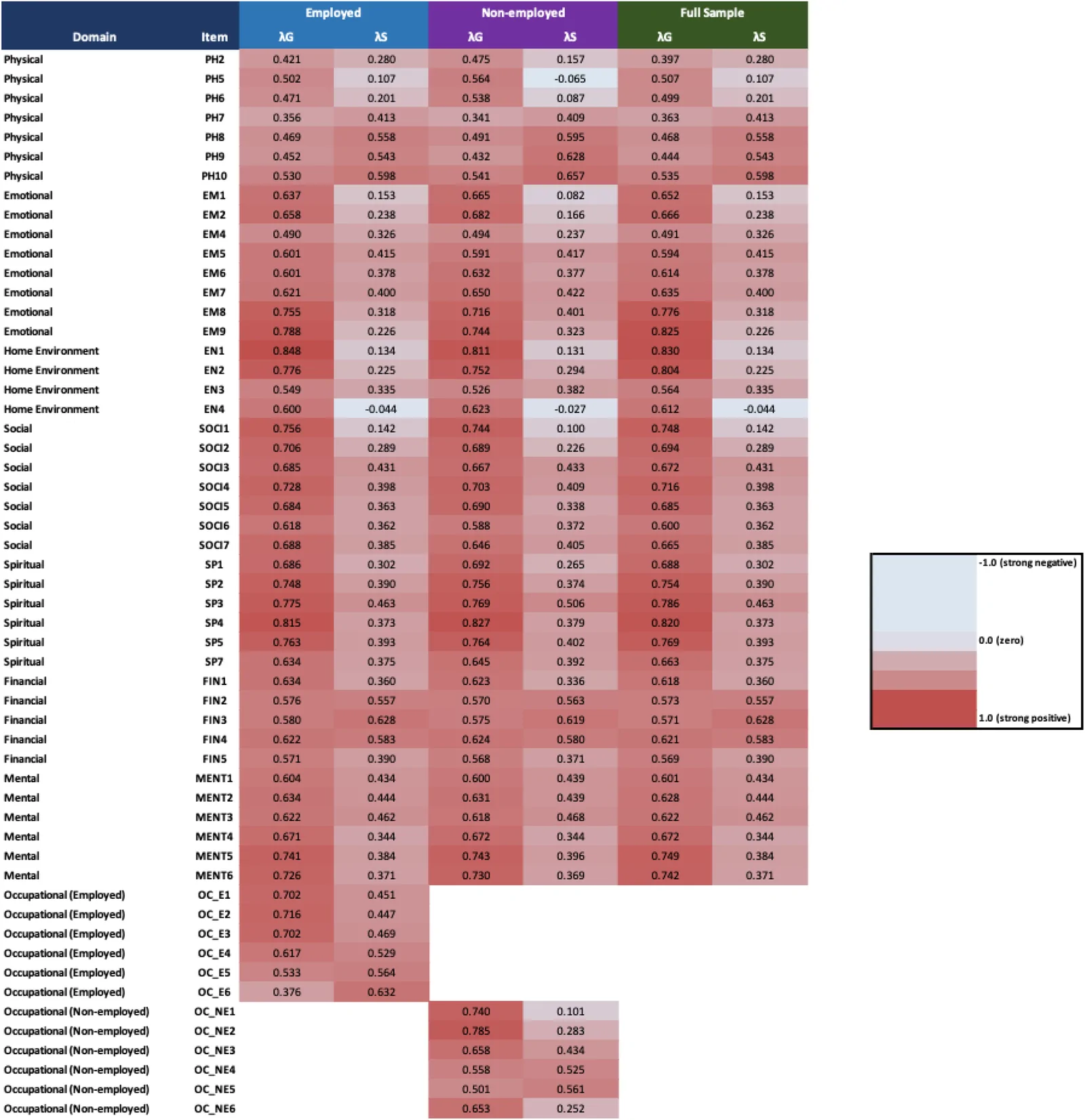
Bifactor ESEM standardized factor loadings (general wellness G-factor and specific domain factors) for the HER WELL scale across employed, non-employed, and full samples.

The general women's wellness factor (G-factor) showed strong and highly consistent performance across all groups (Mean G: Employed=0.637, Non-employed=0.637, Full Sample=0.640), indicating that the overarching wellness construct is stable across employment status. All items loaded significantly on the G-factor (*λ* = 0.341–0.848), with the highest loadings observed for EN1 (Home Environmental), SP4 (Spiritual), and EM9 (Emotional), highlighting these as core indicators of overall wellness. The lowest G-factor loadings were observed for PH7 (Physical) and OC_E6 (Occupational–Employed), suggesting these items capture more domain-specific rather than general wellness variance. See Figure 3.

Specific factor loadings indicated that several domains retained meaningful unique variance beyond the G factor. Within Physical wellness, PH8–PH10 exhibited the strongest specific loadings, whereas PH5 was non-significant in both groups, indicating limited unique physical variance for this item. Emotional items EM5, EM7, and EM8, and Financial items—especially FIN3—showed relatively strong specific loadings, while Emotional item EM1 and Home Environmental item EN4 were weak or non-significant, consistent with these items functioning primarily as expressions of general wellness. Social (SOCI3–SOCI4), Spiritual (notably SP3), and Mental items (especially MENT3) showed moderate specific contributions, whereas Home Environmental items overall displayed the weakest specific variance, reinforcing their role as indicators of global rather than narrowly defined Home Environmental wellness.

To provide a domain-level summary of these item-level bifactor loadings, Table 7 presents the mean G-factor and primary specific loadings for each wellness domain across employed, non-employed, and full samples. As shown in Table 7, Spiritual, Home Environmental, and Social wellness exhibited the highest mean loadings on the general factor (Mean G ≈.69–.75), indicating that these domains are the strongest reflections of overall wellness, whereas Financial, Mental, Physical, and Occupational wellness showed the highest mean specific loadings (≈.38–.52), suggesting they contribute the greatest unique variance beyond general wellness. Differences in mean G between employed and non-employed women were minimal (|*Δ*G| ≤ 0.03 for all domains), supporting structural similarity across groups.

**Table 7:** Mean G-factor and primary specific loadings by domain – three-group comparison.

Domain	n Items	Emp Mean G	Une Mean G	Full Mean G	Emp Mean Spec	Une Mean Spec	Full Mean Spec	*Δ* G (E−U)
Physical	7	0.457	0.483	0.459	0.386	0.353	0.386	-0.026
Emotional	8	0.644	0.647	0.657	0.307	0.303	0.307	-0.003
Environml	4	0.693	0.678	0.703	0.162	0.197	0.162	+0.015
Social	7	0.695	0.675	0.683	0.339	0.326	0.339	+0.020
Spiritual	6	0.737	0.742	0.747	0.383	0.386	0.383	-0.005
Financial	5	0.597	0.592	0.59	0.504	0.494	0.504	+0.005
Mental	6	0.666	0.666	0.669	0.407	0.409	0.407	+0.000
Occupational (Emp)	6	0.608	—	—	0.515	—	—	—
Occupational (Une)	6	—	0.649	—	—	0.359	—	—

Mean G = mean STDYX loading on G-factor (Women’s Wellness) across domain items. Mean Spec=mean primary specific factor loading. *Δ* G = Employed mean G minus Non-employed mean G.

### Final measurement models quality

To comprehensively evaluate the quality of the final measurement models, indices were derived from both the ESEM and Bifactor ESEM solutions for the HER-WELL scale. The ESEM-based reliability and validity indices quantify how well each wellness domain functions as a distinct, multidimensional subscale, whereas the bifactor ESEM indices additionally decompose score variance into a general wellness factor and domain-specific factors, capturing the hierarchical nature of women's wellness. Evaluating measurement quality at both levels—ESEM for correlated dimensions and bifactor ESEM for the general-plus-specific structure—supports the defensible use of both domain scores and a holistic wellness score in applied work with Saudi women.

Accordingly, the following subsections present:

1. Measurement Quality (ESEM) – internal consistency, convergent validity, and Discriminant validity for the eight wellness domains under the ESEM representation.
2. Measurement Quality (Bifactor ESEM) – bifactor reliability indices (for the general and specific factors) and additional evidence regarding convergent and Discriminant validity under the bifactor ESEM representation

### Measurement quality (ESEM)

#### Reliability

Table 8 presents McDonald's omega (*ω*) and Cronbach's alpha (*α*) for each HER-WELL domain across the employed, non-employed, and full samples. Across all three samples, McDonald's omega (*ω*) and Cronbach's alpha (*α*) values indicated good to excellent internal consistency for most HER-WELL domains (*ω* = 0.767–.909; *α* = 0.742–.899), with the lowest estimates observed for the Home Environmental factor and the highest for Spiritual and Mental wellness. Spiritual, Financial, and Occupational domains showed particularly strong reliability, with *ω* and *α* values consistently above.83 across all samples in which they were estimated.

**Table 8:** Reliability and validity indices for the HER-WELL eight-factor ESEM across employed, Non-employed, and full sample.

**Domain**	**Employed**			**Non-employed**			**Full sample**		
	**ω**	**α**	**AVE**	**ω**	**α**	**AVE**	**ω**	**α**	**AVE**
Physical	0.809	0.807	0.406	0.809	0.808	0.389	0.810	0.808	0.401
Emotional	0.871	0.869	0.309	0.873	0.871	0.281	0.872	0.870	0.254
Home Environmental	0.767	0.745	0.122	0.766	0.742	0.105	0.767	0.744	0.070
Social	0.860	0.859	0.344	0.862	0.861	0.280	0.862	0.861	0.372
Spiritual	0.891	0.871	0.536	0.909	0.899	0.461	0.901	0.888	0.577
Financial	0.831	0.825	0.428	0.850	0.847	0.555	0.844	0.840	0.454
Mental	0.866	0.862	0.331	0.881	0.877	0.310	0.876	0.872	0.358
Occupational	0.866	0.865	0.627	0.845	0.841	0.479	*—*	*—*	*—*

Note. ω = McDonald’s omega (ω₃); α = Cronbach’s alpha; AVE, average variance extracted. Estimator: WLSMV; Parameterization: Theta; Rotation: TARGET (Orthogonal). All indicators treated as categorical.). — = factor not estimated in this group (Occupational factor not included in the Full Samplemodel).

#### Convergent validity

In ESEM, items are permitted to cross-load on non-target factors, which means that item variance is distributed across multiple factors simultaneously; as a result, AVE values are systematically attenuated relative to conventional CFA-based thresholds and should not be interpreted using the traditional ≥.50 benchmark in isolation (20). Accordingly, convergent validity in the present study is primarily established by the dominance of primary factor loadings over cross-loadings, as reported in the previous section. This held for every item with reportable data in Emotional, Financial, Mental, Occupational, and Spiritual wellness (gaps ranging from.02 to.87 depending on domain), with all cross-loadings remaining below the.50 threshold recommended for well-defined ESEM models (44, 46) Physical and Social wellness showed this pattern for most items, with one exception each: PH5 (Physical) showed a primary loading smaller than its largest cross-loading in the Full and Non-employed samples, and Soci1 (Social) showed the same pattern in the Non-employed sample. For Home Environmental wellness, this pattern did not hold: in the Full sample, three of four items (EN1, En2, En4) showed a primary loading smaller than their largest cross-loading, and En3 showed the same pattern in the Non-employed sample.

Supplementary AVE values are reported in Table 8 for transparency. Only the Spiritual (AVE =.461–.577) and Financial domains in the non-employed sample (AVE =.555), and the Occupational factor in the employed sample (AVE =.627), met the conventional ≥.50 threshold, while the remaining domains showed attenuated AVE values consistent with the cross-loading structure inherent to ESEM. These results should be interpreted alongside the primary loading pattern in the Factor Loadings and Cross-Loadings section, which collectively provides sufficient evidence of convergent validity at the item level.

#### Discriminant validity

Discriminant validity was evaluated using the Heterotrait–Monotrait ratio of correlations (HTMT; Henseler et al., 2015), with values below.85 indicating adequate discriminant validity and values below.90 representing an alternative lenient threshold. As shown in Table 9, Most HTMT values fell below the conservative.85 threshold, indicating acceptable discriminant validity. A few conceptually adjacent pairs (e.g., Emotional–Home Environment and Home Environment–Social) exceeded.85 but remained below the.90 lenient cutoff, suggesting expected overlap between conceptually related domains without severe discriminant-validity violations. Crucially, no factor pair reached HTMT ≥.90 in any sample, indicating that all eight HER-WELL dimensions retain sufficient discriminant validity to be treated as related but non-redundant facets of women's holistic wellness.

**Table 9:** Discriminant validity for the HER-WELL eight-factor ESEM across employed, Non-employed, and full sample.

Discriminant Validity (HTMT) — Employed								
Factor	Physical	Emotional	Environ.	Social	Spiritual	Financial	Mental	Occup.
Physical	—	0.693	0.596	0.597	0.426	0.586	0.565	0.469
Emotional	0.693	—	0.851	0.821	0.686	0.604	0.709	0.487
Home Environmental	0.596	0.851	—	0.864	0.786	0.650	0.632	0.484
Social	0.597	0.821	0.864	—	0.801	0.680	0.745	0.574
Spiritual	0.426	0.686	0.786	0.801	—	0.636	0.564	0.515
Financial	0.586	0.604	0.650	0.680	0.636	—	0.788	0.551
Mental	0.565	0.709	0.632	0.745	0.564	0.788	—	0.585
Occupational	0.469	0.487	0.484	0.574	0.515	0.551	0.585	—

Discriminant Validity (HTMT) — Non-employed								
Factor	Physical	Emotional	Environ.	Social	Spiritual	Financial	Mental	Occup.
Physical	—	0.761	0.703	0.668	0.590	0.659	0.675	0.668
Emotional	0.761	—	0.862	0.859	0.761	0.580	0.755	0.743
Home Environmental	0.703	0.862	—	0.833	0.774	0.566	0.649	0.747
Social	0.668	0.859	0.833	—	0.831	0.639	0.787	0.765
Spiritual	0.590	0.761	0.774	0.831	—	0.615	0.668	0.735
Financial	0.659	0.580	0.566	0.639	0.615	—	0.763	0.692
Mental	0.675	0.755	0.649	0.787	0.668	0.763	—	0.780
Occupational	0.668	0.743	0.747	0.765	0.735	0.692	0.780	—

Discriminant Validity (HTMT) — Full Sample								
Factor	Physical	Emotional	Environ.	Social	Spiritual	Financial	Mental	
Physical	—	0.731	0.654	0.637	0.513	0.631	0.630	
Emotional	0.731	—	0.858	0.843	0.726	0.593	0.736	
Home Environmental	0.654	0.858	—	0.849	0.780	0.606	0.643	
Social	0.637	0.843	0.849	—	0.818	0.659	0.770	
Spiritual	0.513	0.726	0.780	0.818	—	0.621	0.621	
Financial	0.631	0.593	0.606	0.659	0.621	—	0.778	
Mental	0.630	0.736	0.643	0.770	0.621	0.778	—	

HTMT=Heterotrait–Monotrait ratio of correlations. Values on the diagonal represent the factor itself (set to —). HTMT <.85 indicates adequate discriminant validity (Henseler et al., 2015); HTMT <.90 is an alternative lenient criterion (Gold et al., 2001). Estimator: WLSMV; Parameterization: Theta; Rotation: TARGET (Orthogonal). All indicators treated as categorical. The Occupational factor was not estimated in the All Women model (—).

It is further noted that HTMT values in ESEM are typically lower than those obtained from equivalent ICM-CFA models, because the estimation of cross-loadings prevents factor correlations from being inflated by misattributed item variance (20). The HTMT pattern observed here, therefore, provides a realistic and conservative assessment of discriminant validity within the ESEM framework.

Together, the reliability and validity indices derived from the ESEM solution confirm that the HER-WELL scale yields consistent domain-level scores, with empirically distinguishable factors despite theoretically meaningful intercorrelations. The next section extends this evaluation to the Bifactor ESEM solution, which additionally partitions variance into a general wellness factor and domain-specific components.

### Bifactor measurement quality (global and factor-level)

#### Global bifactor indices

Global bifactor indices for the general wellness factor (G) are summarised in Table 10. Unlike standard CFA factors, G in the bifactor ESEM model does not represent a directly measured latent construct with its own dedicated items. Rather, it captures the shared variance across all eight wellness domains, reflecting a broad general wellness disposition that underlies domain-specific wellness experiences. The pattern of indices provided strong support for the meaningfulness and precision of this general factor across employment groups.

**Table 10:** Bifactor ESEM quality criteria indices across employed, Non-employed, and full sample groups.

Index	Value	Value	Value	Criterion
ECV — Explained Common Variance	0.549	0.655	0.623	≥ 0.50
ω — McDonald's Omega	0.982	0.983	0.980	≥ 0.90
ωh — Omega Hierarchical	0.884	0.939	0.918	≥ 0.80
H — Construct Reliability (H)	0.970	0.975	0.970	≥ 0.80
FD — Factor Determinacy	0.986	0.987	0.985	≥ 0.90

ECV=Explained Common Variance (proportion of total common variance explained by the general factor; adequate ≥.50). ω = McDonald’s Omega (total scale reliability). ωh = Omega Hierarchical (reliability attributable to the general factor; adequate ≥.80). H = Construct Reliability index (maximal reliability of the latent factor; adequate ≥.80). FD = Factor Determinacy (accuracy of factor score estimation; adequate ≥.90). Gold-shaded cells (▪) indicate values meeting or exceeding the recommended threshold. Estimator: WLSMV; Parameterization: Theta; Rotation: TARGET (Orthogonal). All indicators treated as categorical.

ECV values ranged from.549 to.655, indicating that between 55% and 66% of the common variance is attributable to G exceeding the ≥.50 adequacy threshold recommended for bifactor models in which subscales are theoretically retained (19). We do not apply the more stringent ≥.70 threshold here, as this was proposed by Reise et al., (2013). Specifically for predicting bias when misspecifying a multidimensional model as unidimensional — not the present case, where the bifactor structure is retained, and subscale scores are reported alongside the total score. ECV values in the.50–.70 range are consistent with a strong yet genuinely multidimensional construct, as observed in comparable bifactor ESEM validation studies (60, 61).

McDonald's omega was exceptionally high across all groups (*ω* = 0.982–.983),and omega hierarchical values (*ω*H = 0.884–.939) confirmed that most of this reliability is attributable to G. Construct replicability (H = 0.970–.975) and factor determinacy coefficients (FD = 0.985–.987) were well above recommended thresholds (H ≥ 0.80; FD ≥ 0.90 (62);, supporting the validity of the total HER-WELL score as a precise index of general wellness.

### Specific-Factor indices and unique variance

Factor-level bifactor indices are reported in Table 11. ECV_SS values ranged from.135 to.546, highest for Occupational wellness in the employed group (.546) and for Physical and Financial wellness in the full sample (.500 and.311). Omega for specific factors was uniformly high (*ω* = 0.846–.961), but omega hierarchical (*ω*hs = 0.14–.57) was generally moderate an expected pattern in bifactor models of broad psychological constructs (59, 62).

**Table 11:** Factor-Level bifactor ESEM quality criteria across employed, Non-employed, and full sample groups.

	Employed							Non-employed							Full Sample						
Factor	ECV_SS	ECV_SG	ECV_GS	ω	ωh	H	FD	ECV_SS	ECV_SG	ECV_GS	ω	ωh	H	FD	ECV_SS	ECV_SG	ECV_GS	ω	ωh	H	FD
G (General)	0.549	0.549	0.549	0.982	0.884	0.970	0.986	0.655	0.655	0.655	0.983	0.939	0.975	0.987	0.623	0.623	0.623	0.980	0.918	0.970	0.985
Physical	0.365	0.061	0.354	0.896	0.379	0.710	0.757	0.443	0.054	0.392	0.888	0.437	0.694	0.730	0.500	0.062	0.331	0.885	0.473	0.678	0.704
Emotional	0.140	0.066	0.529	0.951	0.183	0.649	0.602	0.227	0.036	0.690	0.891	0.215	0.490	0.494	0.172	0.050	0.561	0.918	0.187	0.552	0.546
Home Environmental	0.135	0.043	0.583	0.951	0.144	0.490	0.618	0.244	0.037	0.603	0.847	0.234	0.460	0.568	0.194	0.039	0.607	0.926	0.181	0.367	0.515
Social	0.148	0.050	0.565	0.961	0.167	0.621	0.654	0.199	0.036	0.708	0.916	0.192	0.508	0.518	0.247	0.050	0.676	0.909	0.235	0.563	0.566
Spiritual	0.225	0.047	0.489	0.957	0.244	0.612	0.560	0.184	0.040	0.688	0.955	0.188	0.545	0.565	0.235	0.049	0.586	0.936	0.248	0.569	0.579
Financial	0.268	0.045	0.553	0.899	0.271	0.583	0.635	0.338	0.049	0.519	0.918	0.329	0.665	0.679	0.311	0.058	0.532	0.917	0.300	0.634	0.658
Mental	0.238	0.061	0.565	0.931	0.250	0.642	0.674	0.217	0.056	0.676	0.932	0.214	0.578	0.555	0.270	0.069	0.595	0.926	0.275	0.612	0.569
Occupational	0.546	0.080	0.330	0.929	0.565	0.838	0.863	0.295	0.039	0.652	0.884	0.261	0.551	0.636	*—*	*—*	*—*	*—*	*—*	*—*	*—*

Note. ECV_SS = Explained Common Variance for the specific factor (subscale-level); ECV_SG = ECV of the specific factor relative to the general factor; ECV_GS = ECV of the general factor relative to the specific factor. ω = McDonald’s Omega (total reliability); ωh = Omega Hierarchical (reliability due to the general factor; adequate ≥.80); H = Construct Reliability (adequate ≥.80); FD = Factor Determinacy (adequate ≥.90). ECV_SS adequate ≥.50. Gold-shaded cells (▪) indicate values meeting or exceeding the recommended threshold. G = general factor row. — = factor not estimated in this group (Occupational not included in Full Sample model). Estimator: WLSMV; Parameterization: Theta; Rotation: TARGET (Orthogonal).

Physical, Financial, Mental, and Occupational wellness displayed the strongest domain-specific reliability (*ω*hs = 0.21–.57; H = 0.55–.86), retaining sufficient unique variance to support interpretation of their subscale scores. Occupational wellness in the employed group was strongest overall (ECV_SS = 0.546, *ω*hs = 0.565, H = 0.838, FD = 0.863). Emotional, Home Environmental, Social, and Spiritual wellness showed lower *ω*hs (.14–.25) and H (.37–.65), indicating these domains are more heavily saturated by G and should be interpreted with appropriate caution as subscale scores.

Taken together, these indices indicate that HER-WELL is characterized by a strong, dominant general wellness factor that supports a highly reliable total score, while Physical, Financial, Mental, and Occupational wellness retain sufficient unique variance to justify reporting subscale scores alongside it. Emotional, Home Environmental, Social, and Spiritual wellness are more strongly intertwined with the general factor, primarily reflecting holistic wellness rather than differentiated facets in this sample. Accordingly, subsequent analyses prioritize the HER-WELL total score, with domain scores providing complementary descriptive profiles.

### Item retention and subscale scoring

Item retention and subscale-scoring decisions were governed by two distinct criteria applied to the retained bifactor ESEM model. First, items were retained in the overall wellness score based on their general-factor (G) loading, which all retained items exceeded (*λ*G ≥ 0.44 for PH2/PH5; *λ*G = 0.58–.85 for Home Environment items), confirming their contribution to the total HER-WELL score regardless of domain-specific performance. Second, whether a domain supports an independent subscale score was evaluated using factor-level bifactor indices (Table 11). Physical, Financial, Mental, and Occupational wellness met or approached recommended thresholds for specific-factor reliability and replicability (H = 0.55–.86; *ω*HS = 0.21–.57) and are reported as interpretable subscale scores, with appropriate caution where indices are borderline. Emotional, Home Environment, Social, and Spiritual wellness did not meet these thresholds (H = 0.37–.65; *ω*HS = 0.14–.25), with Home Environment showing the weakest specific-factor indices of any domain (H = 0.367–.490; ECV_SS = 0.135–.244), consistent with its weak convergent validity (AVE =.07–.12). Accordingly, we do not recommend that Emotional, Home Environment, Social, or Spiritual wellness be scored or interpreted as independent subscales; these domains should be interpreted only through the HER-WELL total score.

## Discussion

To our knowledge, the HER-WELL scale is the first comprehensive, multidimensional wellness instrument to be psychometrically validated specifically for Saudi women, with potential applicability to Arab women in similar socio-cultural contexts. Previous Arabic instruments for Saudi women have focused on more specific constructs such as psychological empowerment, family stability, stigma, or condition-specific quality of life—rather than providing a broad, integrated assessment of women's wellness across multiple life domains (63–66).

Existing wellness instruments, such as the Wellness Inventory, TestWell Wellness Inventory, Wellness Evaluation of Lifestyle, Perceived Wellness Scale, Five Factor Wellness Inventory, and related tools, were all developed in Western, predominantly mixed-gender or general-population samples and were not designed to capture gendered or context-specific dimensions of women's wellness (1–4, 67, 68). These measures, therefore, give limited attention to domains central to the lived experiences of Saudi and Arab women, including religious practice, family and caregiving roles, and the Home Environment, and have rarely undergone rigorous psychometric testing in non-Western female populations. A recent systematic review of wellness models identified thirteen theoretical frameworks, all predominantly rooted in Western contexts, underscoring the pressing need for culturally sensitive and gender-responsive wellness measurement tools (69). By contrast, HER-WELL was developed in Arabic, explicitly grounded in the Eight Dimensions of Wellness framework, refined through qualitative work with Saudi women, and subsequently validated in a large, stratified national sample using contemporary psychometric methods (ESEM, bifactor ESEM, WLSMV, and Dynamic Fit Index cutoffs), demonstrating strong internal consistency, a dominant general wellness factor, and interpretable domain-specific scores.

Among the systematic comparisons of the six models, the ESEM and bifactor ESEM solutions provided the best fit in the employed, non-employed, and full samples, with the bifactor ESEM emerging as the most adequate representation of the data. The bifactor ESEM model achieved excellent global fit indices (CFI ≈ 0.985–0.988, TLI ≈ 0.977–0.981, RMSEA ≈ 0.029–0.032, SRMR ≈ 0.020–0.024) that exceeded both conventional and DFI-tailored cutoffs. This superiority is not merely statistical but theoretically meaningful. Wellness is a multidimensional phenomenon whose domains are conceptually related yet empirically distinct, a structure that conventional ICM-CFA, with its requirement of zero cross-loadings, systematically fails to represent (70). ESEM and bifactor ESEM are particularly well-suited to such constructs, as they accommodate the inherent item complexity that arises when Likert-scaled items tap into more than one latent dimension simultaneously. The bifactor ESEM framework resolves this by partitioning variance into a general wellness factor and domain-specific factors, while permitting cross-loadings to be freely estimated rather than constrained to zero, yielding a more accurate and unbiased representation of the underlying measurement structure (15, 44, 71). In line with this, Alamer, (2022) similarly reported that bifactor ESEM outperformed bifactor CFA in goodness-of-fit owing to its greater analytical flexibility, and that CFA solutions failed to adequately represent the measurement model across independent samples.

Several items demonstrated modest primary factor loadings in the ESEM solution (ranging from.201 to.499), particularly within the Physical and Home Environmental domains. Consistent with Alamer, (2022) guidelines, loadings between.30 and.50 were retained in the total wellness score on the basis of their general-factor (*λ*G) loadings and their contribution to the bifactor ESEM general factor, rather than their domain-specific loadings alone. Four items [PH2, PH5, EN4, Soci1(Non-employed sample)] showed loadings below.30 in some analytical conditions and are flagged for re-examination or revision in future validation studies.

The Home Environment domain's AVE was critically low across all three samples (.070–.122), with three of four items (EN2, EN3, EN4) showing primary loadings below.30 in the full-sample ESEM solution (range:.201–.352). Together with the factor-level bifactor indices reported in Table 11 (H = 0.367–.490; *ω*HS = 0.135–.244), these values confirm that the specific Home Environment factor captures minimal unique variance beyond the general wellness factor and does not meet conventional standards for a psychometrically distinct subscale. As stated in the Results, we therefore do not recommend scoring or interpreting the Home Environment domain as a stand-alone subscale; its items should be retained only as contributors to the general wellness composite. This limitation, alongside the domain's conceptual breadth (encompassing both physical and social environmental factors), suggests that future revisions of HER-WELL should consider redeveloping or replacing the Home Environment items to yield a more psychometrically coherent domain-specific measure.

Within the bifactor-ESEM framework, the general wellness factor emerged as clearly dominant, capturing the majority of common variance across items and exhibiting excellent reliability and replicability indices across all groups. ECV values ranged from approximately.55 to.66, indicating that more than half of the common variance among HER-WELL items is attributable to the general wellness factor, levels that are typically interpreted as evidence of a substantively important general factor in theoretically multidimensional bifactor scales (62, 72). Rather than debating stricter ECV benchmarks, these values are best understood as indicating that a strong general factor coexists with meaningful multidimensionality, consistent with the theoretical design of HER-WELL to retain distinct wellness domains alongside an overall score (18).

The primary psychometric justification for the total score rests instead on the omega hierarchical coefficients [*ω*H = 0.884–.939, all exceeding the ≥.80 recommended threshold; Reise et al., (2013)], which confirm that the large majority of total score variance is directly attributable to general wellness rather than to domain-specific factors. This finding is further corroborated by outstanding construct replicability (H = 0.970–.975) and factor determinacy coefficients (FD = 0.985–.987), both well above recommended thresholds [H ≥ 0.80; FD ≥ 0.90; Rodriguez et al., (2016a)], and by total composite reliability (*ω* = 0.980–.983), confirming that HER-WELL functions primarily as a robust, well-defined general wellness measure. In this context, the domain-specific factors contribute targeted residual information beyond the general factor rather than constituting fully independent scales. In applied settings, the total HER-WELL score functions as the primary screening indicator of holistic wellness; however, when a woman presents with a low total score, domain profiles, particularly financial, occupational, mental, and physical wellness, can guide targeted intervention. Because these domains retain meaningful specific-factor variance beyond the general wellness factor, their scores carry information that the total score alone does not fully capture. For example, two women with identical total scores may present with markedly different domain profiles: one may show concentrated financial and occupational strain, which we hypothesize could indicate the need for economic support and work-related interventions, while the other may show deficits primarily in physical and mental wellness, potentially suggesting relevance for health promotion and psychological services. These are illustrative possibilities rather than demonstrated applications; as noted below, systematic tests of HER-WELL's predictive and criterion validity are needed before domain profiles can guide actual intervention decisions. By contrast, spiritual, social, and Home Environment wellness, which are more tightly integrated with the general factor, are less useful as independent intervention targets but can be interpreted as reflecting the overall wellness context in which specific-domain deficits occur.

These findings align with recent work on the Wellness Inventory, in which exploratory and confirmatory factor analyses supported a single-factor solution and led to the interpretation of wellness as a holistic, unidimensional construct despite an eight-dimensional conceptual model (1). In line with this work, HER-WELL is best interpreted primarily through a general wellness score; however, by adopting a bifactor ESEM approach, we also demonstrate that women's wellness is hierarchically organized rather than strictly unidimensional.

Beyond the dominant general factor, the specific wellness domains retain a secondary but practically meaningful role. Domain-level bifactor indices show that occupational wellness (among employed women) and, to a lesser extent, physical, financial, and mental wellness exhibit comparatively higher specific-factor ECV_SS, *ω*h, and H values—for example, the occupational factor in employed women exceeds the recommended ECV_SS ≥ 0.50 guideline and shows strong construct replicability (H ≈.84), while the physical, financial, and mental factors display consistently higher specific-factor indices than the remaining domains. This pattern indicates that these subscales capture unique wellness resources and challenges that are not fully absorbed by the general factor, which is consistent with bifactor guidelines where non-trivial ECV_SS, *ω*h, and H values are interpreted as evidence of construct-relevant multidimensionality rather than mere nuisance residuals (44, 62). In contrast, spiritual, social, and Home Environment wellness show lower specific-factor ECV_SS and *ω*h values and more modest H coefficients, while simultaneously displaying particularly strong loadings on the general factor, suggesting that in this context these domains function primarily as core expressions of general wellness rather than as sharply differentiated subconstructs. This configuration mirrors bifactor applications in which some facets largely define the general factor, whereas others retain more domain-specific information (18, 73). Taken together, these findings support viewing HER-WELL as a general-plus-specific system in which spiritual, social, and Home Environment wellness largely anchor the shared core of wellness, whereas physical, financial, mental, and especially occupational wellness provide more fine-grained profiles of functional capacity and constraint that can inform targeted intervention.

Lastly, the HER-WELL scale demonstrated strong construct validity across reliability, convergent, and discriminant indicators within the ESEM framework. Domain-level internal consistency was good to excellent (*ω* ≈.77–.91; *α* ≈.74–.90), with particularly high reliability for spiritual, financial, and occupational wellness, comparable to or exceeding that reported for other Arabic instruments developed for Saudi women (e.g., empowerment and wellbeing scales). Convergent validity was supported by the clear dominance of primary over cross-loadings, with all items loading significantly on their intended domains and several domains (especially spiritual, financial, and occupational wellness) showing consistently strong primary loadings, in line with recommendations to prioritize loading patterns over AVE in ESEM, where cross-loadings systematically attenuate AVE values (20, 44). Discriminant validity, evaluated via the Heterotrait–Monotrait ratio, indicated that most factor pairs fell well below the.85 guideline, with the highest HTMT values observed for conceptually adjacent pairs such as Emotional–Home Environmental and Home Environmental–Social wellness, reflecting meaningful theoretical overlap rather than redundancy; critically, no HTMT value reached.90, confirming that the eight domains remain empirically distinguishable facets of women's wellness (74). Taken together, these reliability and validity indices indicate that HER-WELL yields consistent, convergent, and non-redundant domain scores within a realistic ESEM representation, thereby justifying the use of both the total and subscale scores in research and applied settings.

Relative to traditional wellness instruments developed in Western populations, mixed-gender populations are often estimated using ICM-CFA with zero cross-loadings and evaluated against generic fit thresholds. HER-WELL's use of ESEM, bifactor ESEM, and Dynamic Fit Index-based cutoffs represents a substantive methodological contribution, offering a more realistic representation of interrelated wellness dimensions and more rigorous evidence of construct validity for women in a non-Western setting. Compared to other recent well-being scale validations, Faleh et al., (2025) developed the Obesity-Related Well-Being scale using conventional EFA/CFA without modeling cross-loadings or a general factor, limiting direct methodological comparison (75). A closer parallel is Olefir et al., (2025) who compared CFA, bifactor CFA, ESEM, and bifactor ESEM models of subjective well-being (N = 1,111) using the same four-model comparison logic employed here; their bifactor ESEM was decisively preferred (AICc weight=1.00) and revealed a dominant general factor (ECV =.63; *ω*H = 0.72) alongside comparatively weaker specific factors (ECV_specific=15%–51%), a pattern consistent with though somewhat less pronounced than HER-WELL's general factor saturation (ECV =.549–.655; *ω*H = 0.884–.939) (76).

### Theoretical and practical implications

The bifactor-ESEM results corroborate these contributions by empirically demonstrating a strong general wellness factor alongside culturally specific domains that reflect Saudi women lived experiences. This study makes several important theoretical contributions. First, it conceptualises women's wellness as a complex, multidimensional yet hierarchically organised construct, recognising that its domains are interconnected in ways unique to women's experiences rather than reducible to a single score or to fully independent subscales. Second, the research adapts and extends existing wellness frameworks to explicitly centre what matters to women in a Saudi cultural context, grounded in qualitative evidence from Saudi women themselves. Third, by examining the intersection of gender and culture, the study demonstrates that women's wellness cannot be fully understood through Western or gender-neutral frameworks alone. Fourth, the psychometric validation of HER-WELL with a large, stratified national sample lays an empirical foundation for future studies in Arab and similar cultural contexts. Together, these contributions offer a culturally conscious and methodologically rigorous approach to measuring women's wellness, challenging the assumption that Western-derived frameworks apply equally across all settings.

### Limitations and future directions

Despite these strengths, several limitations should be considered when interpreting the present findings. First, the sample was drawn exclusively from Saudi Arabian women, which, while appropriate for the scale development purpose, constrains the generalisability of HER-WELL beyond this population. Although the scale may have theoretical relevance for Arab women in similar socio-cultural contexts, its psychometric properties and factor structure have not been tested in other Arabic-speaking or Muslim-majority populations. Future studies should examine the cross-cultural portability of HER-WELL through translation, adaptation, and validation in other Arab and non-Western settings.

Second, although data were collected via structured phone interviews, a mode that reduces concerns about item misunderstanding and low engagement compared with self-administered formats this approach is not immune to information bias, particularly social desirability bias (77). Social desirability bias arises when participants adjust their responses in the presence of an interviewer, especially when questions concern personal or sensitive topics such as emotional well-being, financial security, or family dynamics. Several design measures were implemented to mitigate this risk: participants were randomly selected, anonymity and confidentiality were assured at the outset of each interview, interviewers followed a standardized protocol, and a five-point Likert response format was used to provide sufficient response variability without forcing extreme endorsement. Collinearity diagnostics further indicated that common method bias is unlikely to be severe (VIFs < 3.3) (78). Nonetheless, future studies might complement interviewer-administered data with self-administered formats or qualitative follow-up interviews to examine whether response patterns differ across modes and to further validate the construct meanings as understood by participants.

Thirdly, some domain-level specific factors (particularly emotional and Home Environmental wellness) displayed limited construct replicability. Home Environmental domain in particular showed critically low AVE (.070–.122) and weak specific-factor loadings, indicating it does not function as a statistically distinct construct within this sample and should not be scored or interpreted as an independent subscale. This is consistent with the primary design intent of HER-WELL, which was developed to provide a valid overall measure of women's holistic wellness rather than a battery of independent domain-specific instruments; these domains are therefore most meaningfully interpreted as core expressions of general wellness. Researchers who require precise, independent measurement of a single wellness domain should use dedicated domain-specific scales. Future work could also explore whether a shortened version of HER-WELL, retaining items with strong loadings on the general wellness factor and representing core content within each domain such as (EN1, SP4, EM9), achieves comparable psychometric performance while offering greater brevity for large-scale surveys or routine screening.

Lastly, three further limitations warrant note. Measurement invariance across employment status was not formally tested (only descriptive comparison of general-factor loadings), so equivalence of the HER-WELL structure across these groups remains unconfirmed. The Spiritual Wellness domain showed a pronounced ceiling effect, limiting its ability to discriminate among respondents at the upper end of the construct. Finally, the reliability evidence here reflects only internal consistency; no test-retest coefficient was obtained, so temporal stability is unknown. Establishing measurement invariance and test-retest reliability are priorities for the next phase of validation.

Moreover, the present study focused primarily on substantive and structural aspects of validity; systematic tests of HER-WELL within a broader nomological network and examinations of predictive validity were beyond its scope (79). This limitation bears directly on the applied domain-profile illustrations offered above, which should be read as hypotheses to be tested rather than as validated clinical or programmatic guidance. Future longitudinal and external-validation studies should therefore investigate how HER-WELL scores relate to theoretically linked constructs (e.g., social support, psychological distress, life satisfaction) and the extent to which they predict important health and well-being outcomes among Saudi women over time and across settings.

## Conclusion

The HER-WELL scale represents the first validated, culturally grounded measure of holistic wellness developed specifically for Saudi women. Bifactor ESEM analyses confirmed that HER-WELL is best conceptualized as a hierarchical construct comprising a strong general wellness factor accounting for 55%–66% of common variance and demonstrating excellent reliability (*ω* ≈.98), hierarchical reliability (*ω*_h = 0.88–.94), construct replicability (H ≈.97), and factor determinacy (FD ≈.99) alongside four domain-specific factors (Physical, Financial, Mental, and Occupational wellness) that retained sufficient unique variance for independent subscale interpretation, with the remaining four domains (Emotional, Social, Spiritual, and Home Environmental wellness) more meaningfully interpreted as expressions of the general factor. These psychometric properties support using the HER-WELL total score as the primary outcome for holistic wellness assessment in population health monitoring, intervention evaluation, and policy benchmarking, with domain profiles serving as complementary lenses for targeted support. By grounding measurement in the Eight Dimensions of Wellness framework, in which spiritual items showed the strongest general-factor loadings of any domain, reflecting the centrality of spirituality within Islamic holistic health perspectives, and validating the scale in a large national sample of Saudi women, HER-WELL provides a robust and culturally responsive foundation for longitudinal wellness research, cross-cultural adaptation, and evidence-based wellness promotion across Saudi Arabia and the broader Arab region.

## Data Availability

The data supporting the findings of this study are available from the corresponding author upon reasonable request.
