# Supplementary material for: Construct validation of HER-WELL: a bifactor exploratory structural equation modeling evaluation of an eight-domain wellness scale for women
Source: Front Glob Womens Health. 2026 Aug 18;7:1903478. doi: 10.3389/fgwh.2026.1903478 (PMC13529770; doi:10.3389/fgwh.2026.1903478)
Supplement: Supplementary file 1 [file Table1.docx]

**Appendix 1: Descriptive statistics of Employed, Non‑employed, full sample.**

| **Item** | **Employed** | | | | | **Non‑employed** | | | | | **Full sample** | | | | |
| --- | --- | --- | --- | --- | --- | --- | --- | --- | --- | --- | --- | --- | --- | --- | --- |
|  | **Mean** | **Mdn** | **SD** | **Skew** | **Kurt** | **Mean** | **Mdn** | **SD** | **Skew** | **Kurt** | **Mean** | **Mdn** | **SD** | **Skew** | **Kurt** |
| **Physical Wellness (10 items)** | | | | | | | | | | | | | | | |
| **Ph1** | 3.18 | 3 | 1.18 | -0.09 | -0.75 | 2.91 | 3 | 1.21 | 0.16 | -0.68 | 3.05 | 3 | 1.19 | 0.04 | -0.77 |
| **Ph2** | 3.48 | 3 | 1.06 | -0.17 | -0.64 | 3.53 | 3 | 1.02 | -0.15 | -0.46 | 3.44 | 3 | 1.06 | -0.16 | -0.60 |
| **Ph3** | 3.79 | 4 | 1.08 | -0.55 | -0.48 | 3.76 | 4 | 1.05 | -0.46 | -0.48 | 3.70 | 4 | 1.11 | -0.45 | -0.64 |
| **Ph4** | 4.29 | 5 | 1.29 | -1.63 | 1.22 | 4.19 | 5 | 1.45 | -1.46 | 0.43 | 4.25 | 5 | 1.34 | -1.54 | 0.86 |
| **Ph5** | 4.58 | 5 | 0.79 | -1.89 | 3.10 | 4.56 | 5 | 0.77 | -1.69 | 2.36 | 4.50 | 5 | 0.86 | -1.72 | 2.36 |
| **Ph6** | 3.76 | 4 | 1.09 | -0.46 | -0.66 | 3.84 | 4 | 1.06 | -0.47 | -0.69 | 3.76 | 4 | 1.10 | -0.44 | -0.73 |
| **Ph7** | 3.22 | 3 | 1.35 | -0.23 | -1.08 | 2.91 | 3 | 1.34 | 0.18 | -1.10 | 3.09 | 3 | 1.34 | -0.04 | -1.11 |
| **Ph8** | 3.44 | 3 | 1.24 | -0.32 | -0.83 | 3.26 | 3 | 1.31 | -0.10 | -1.09 | 3.36 | 3 | 1.27 | -0.20 | -1.01 |
| **Ph9** | 3.60 | 4 | 1.31 | -0.50 | -0.90 | 3.28 | 3 | 1.36 | -0.20 | -1.11 | 3.45 | 3 | 1.34 | -0.33 | -1.08 |
| **Ph10** | 3.78 | 4 | 1.24 | -0.62 | -0.75 | 3.53 | 4 | 1.35 | -0.44 | -1.02 | 3.69 | 4 | 1.28 | -0.57 | -0.83 |
| **Emotional Wellness (9 items)** | | | | | | | | | | | | | | | |
| **Em1** | 4.17 | 4 | 0.98 | -0.97 | 0.21 | 4.05 | 4 | 1.02 | -0.89 | 0.19 | 4.07 | 4 | 1.03 | -0.86 | -0.02 |
| **Em2** | 4.10 | 4 | 0.99 | -0.87 | 0.08 | 3.95 | 4 | 1.07 | -0.66 | -0.45 | 4.01 | 4 | 1.06 | -0.80 | -0.20 |
| **Em3*** | 2.51 | 2 | 1.24 | 0.44 | -0.70 | 2.62 | 3 | 1.18 | 0.28 | -0.72 | 2.56 | 3 | 1.24 | 0.388 | -0.745 |
| **Em4** | 3.95 | 4 | 1.11 | -0.71 | -0.58 | 3.74 | 4 | 1.15 | -0.53 | -0.72 | 3.78 | 4 | 1.17 | -0.57 | -0.73 |
| **Em5** | 3.92 | 4 | 1.05 | -0.61 | -0.45 | 3.90 | 4 | 1.09 | -0.63 | -0.51 | 3.88 | 4 | 1.09 | -0.57 | -0.62 |
| **Em6** | 4.22 | 5 | 0.97 | -1.15 | 0.71 | 4.20 | 5 | 0.99 | -1.12 | 0.57 | 4.17 | 5 | 1.02 | -1.10 | 0.51 |
| **Em7** | 4.11 | 4 | 1.04 | -0.94 | 0.05 | 4.28 | 5 | 1.01 | -1.40 | 1.45 | 4.13 | 5 | 1.07 | -1.08 | 0.38 |
| **Em8** | 4.27 | 5 | 0.99 | -1.22 | 0.77 | 4.39 | 5 | 0.86 | -1.31 | 0.99 | 4.26 | 5 | 0.98 | -1.19 | 0.69 |
| **Em9** | 4.29 | 5 | 0.99 | -1.31 | 0.98 | 4.32 | 5 | 0.94 | -1.36 | 1.38 | 4.25 | 5 | 1.02 | -1.29 | 0.96 |
| **Home Environment Wellness (4 items)** | | | | | | | | | | | | | | | |
| **En1** | 4.50 | 5 | 0.83 | -1.60 | 1.78 | 4.50 | 5 | 0.82 | -1.63 | 2.25 | 4.46 | 5 | 0.88 | -1.58 | 1.77 |
| **En2** | 4.29 | 5 | 0.98 | -1.19 | 0.48 | 4.27 | 5 | 0.97 | -1.22 | 0.86 | 4.26 | 5 | 1.00 | -1.21 | 0.62 |
| **En3** | 3.98 | 4 | 1.21 | -0.98 | -0.07 | 3.70 | 4 | 1.26 | -0.56 | -0.81 | 3.85 | 4 | 1.26 | -0.80 | -0.45 |
| **En4** | 4.38 | 5 | 0.95 | -1.42 | 1.12 | 4.36 | 5 | 0.97 | -1.50 | 1.70 | 4.33 | 5 | 1.00 | -1.41 | 1.15 |
| **Social Wellness (7 items)** | | | | | | | | | | | | | | | |
| **So1** | 4.48 | 5 | 0.86 | -1.64 | 2.13 | 4.50 | 5 | 0.90 | -1.94 | 3.44 | 4.43 | 5 | 0.93 | -1.68 | 2.25 |
| **So2** | 4.25 | 5 | 0.95 | -1.08 | 0.43 | 4.27 | 5 | 0.93 | -1.12 | 0.64 | 4.21 | 5 | 0.99 | -1.11 | 0.50 |
| **So3** | 4.17 | 5 | 1.01 | -1.01 | 0.18 | 4.13 | 4 | 0.97 | -0.85 | -0.20 | 4.12 | 4 | 1.05 | -0.97 | 0.06 |
| **So4** | 4.34 | 5 | 0.91 | -1.36 | 1.43 | 4.27 | 5 | 0.95 | -1.20 | 0.87 | 4.26 | 5 | 0.99 | -1.25 | 0.94 |
| **So5** | 4.29 | 5 | 0.92 | -1.05 | 0.16 | 4.15 | 4 | 0.99 | -0.96 | 0.08 | 4.21 | 5 | 0.98 | -1.06 | 0.39 |
| **So6** | 4.13 | 4 | 0.98 | -0.92 | 0.15 | 4.08 | 4 | 1.00 | -0.84 | 0.04 | 4.08 | 4 | 1.04 | -0.91 | 0.05 |
| **So7** | 4.17 | 4 | 0.94 | -0.92 | 0.14 | 4.15 | 4 | 0.96 | -0.78 | -0.47 | 4.12 | 4 | 0.99 | -0.91 | 0.05 |
| **Spiritual Wellness (7 items)** | | | | | | | | | | | | | | | |
| **Sp1** | 4.56 | 5 | 0.80 | -2.00 | 3.91 | 4.61 | 5 | 0.78 | -2.23 | 4.73 | 4.54 | 5 | 0.85 | -2.01 | 3.68 |
| **Sp2** | 4.74 | 5 | 0.66 | -2.73 | 7.22 | 4.81 | 5 | 0.59 | -3.52 | 12.88 | 4.72 | 5 | 0.72 | -2.83 | 7.92 |
| **Sp3** | 4.74 | 5 | 0.64 | -2.57 | 5.82 | 4.82 | 5 | 0.63 | -4.03 | 17.13 | 4.71 | 5 | 0.74 | -2.82 | 7.74 |
| **Sp4** | 4.74 | 5 | 0.64 | -2.71 | 7.48 | 4.82 | 5 | 0.56 | -3.60 | 13.61 | 4.72 | 5 | 0.69 | -2.77 | 7.71 |
| **Sp5** | 4.69 | 5 | 0.73 | -2.73 | 7.88 | 4.80 | 5 | 0.60 | -3.43 | 12.15 | 4.67 | 5 | 0.76 | -2.67 | 7.25 |
| **Sp6*** | 1.45 | 1 | 0.82 | 1.91 | 3.22 | 1.48 | 1 | 0.86 | 1.87 | 3.20 | 1.49 | 1 | 0.88 | 1.86 | 3.02 |
| **Sp7** | 4.41 | 5 | 0.92 | -1.63 | 2.17 | 4.37 | 5 | 0.92 | -1.34 | 1.00 | 4.35 | 5 | 0.99 | -1.54 | 1.67 |
| **Financial Wellness (5 items)** | | | | | | | | | | | | | | | |
| **Fi1** | 4.18 | 4 | 0.98 | -1.01 | 0.22 | 3.94 | 4 | 1.08 | -0.56 | -0.79 | 4.07 | 4 | 1.06 | -0.86 | -0.23 |
| **Fi2** | 4.28 | 5 | 1.01 | -1.31 | 0.91 | 3.97 | 5 | 1.28 | -0.96 | -0.30 | 4.16 | 5 | 1.13 | -1.19 | 0.42 |
| **Fi3** | 4.05 | 4 | 1.11 | -1.07 | 0.47 | 3.82 | 4 | 1.20 | -0.63 | -0.62 | 3.93 | 4 | 1.18 | -0.84 | -0.22 |
| **Fi4** | 4.37 | 5 | 0.91 | -1.48 | 1.79 | 4.13 | 4 | 1.05 | -1.05 | 0.30 | 4.24 | 5 | 1.03 | -1.32 | 1.03 |
| **Fi5** | 3.86 | 4 | 1.14 | -0.69 | -0.39 | 3.76 | 4 | 1.22 | -0.59 | -0.67 | 3.77 | 4 | 1.21 | -0.62 | -0.63 |
| **Mental Wellness (6 items)** | | | | | | | | | | | | | | | |
| **Me1** | 3.94 | 4 | 0.99 | -0.56 | -0.41 | 3.76 | 4 | 1.14 | -0.54 | -0.51 | 3.82 | 4 | 1.09 | -0.59 | -0.40 |
| **Me2** | 3.88 | 4 | 1.10 | -0.59 | -0.54 | 3.68 | 4 | 1.21 | -0.47 | -0.82 | 3.78 | 4 | 1.16 | -0.55 | -0.67 |
| **Me3** | 3.91 | 4 | 1.13 | -0.82 | -0.12 | 3.85 | 4 | 1.16 | -0.70 | -0.44 | 3.85 | 4 | 1.15 | -0.71 | -0.36 |
| **Me4** | 4.37 | 5 | 0.87 | -1.31 | 1.13 | 4.33 | 5 | 0.91 | -1.28 | 1.06 | 4.27 | 5 | 0.95 | -1.20 | 0.75 |
| **Me5** | 4.30 | 5 | 0.88 | -1.03 | 0.14 | 4.25 | 5 | 0.93 | -1.09 | 0.51 | 4.22 | 5 | 0.95 | -1.06 | 0.48 |
| **Me6** | 4.01 | 4 | 1.04 | -0.82 | -0.01 | 3.99 | 4 | 1.05 | -0.84 | 0.04 | 3.94 | 4 | 1.07 | -0.75 | -0.21 |
| **Occupational Wellness (6 items)** | | | | | | | | | | | | | | | |
| **Oc1** | 4.37 | 5 | 0.95 | -1.39 | 1.03 | 4.30 | 5 | 0.86 | -1.17 | 1.10 | — | — | — | — | — |
| **Oc2** | 3.87 | 4 | 1.21 | -0.76 | -0.46 | 4.32 | 5 | 0.85 | -1.31 | 1.61 | — | — | — | — | — |
| **Oc3** | 3.94 | 4 | 1.17 | -0.88 | -0.12 | 3.93 | 4 | 1.05 | -0.54 | -0.58 | — | — | — | — | — |
| **Oc4** | 4.21 | 5 | 1.02 | -1.11 | 0.45 | 3.69 | 4 | 1.25 | -0.49 | -0.99 | — | — | — | — | — |
| **Oc5** | 3.75 | 4 | 1.17 | -0.47 | -0.79 | 3.62 | 3 | 1.13 | -0.27 | -0.70 | — | — | — | — | — |
| **Oc6** | 4.29 | 5 | 1.00 | -1.35 | 1.15 | 4.17 | 4 | 1.01 | -1.24 | 1.02 | — | — | — | — | — |
|  |  |  |  |  |  |  |  |  |  |  |  |  |  |  |  |
| **Domain-Level Summary (Mean of Item Means ± Mean SD)** | | | | | | | | | | | | | | | |
| **Domain** | **Employed** | |  |  |  | **Non‑employed** | |  |  |  | **Full sample** | |  |  |  |
|  | **Mean** | **SD** |  |  |  | **Mean** | **SD** |  |  |  | **Mean** | **SD** |  |  |  |
| **Physical** | 3.71 | 1.16 |  |  |  | 3.58 | 1.19 |  |  |  | 3.63 | 1.19 |  |  |  |
| **Emotional** | 3.95 | 1.04 |  |  |  | 3.94 | 1.03 |  |  |  | 4.00 | 1.08 |  |  |  |
| **Home Environment** | 4.29 | 0.99 |  |  |  | 4.21 | 1.00 |  |  |  | 4.23 | 1.04 |  |  |  |
| **Social** | 4.26 | 0.94 |  |  |  | 4.22 | 0.96 |  |  |  | 4.21 | 0.99 |  |  |  |
| **Spiritual** | 4.19 | 0.74 |  |  |  | 4.24 | 0.71 |  |  |  | 4.60 | 0.80 |  |  |  |
| **Financial** | 4.15 | 1.03 |  |  |  | 3.92 | 1.17 |  |  |  | 4.03 | 1.12 |  |  |  |
| **Mental** | 4.07 | 1.00 |  |  |  | 3.98 | 1.07 |  |  |  | 3.98 | 1.06 |  |  |  |
| **Occupational** | 4.07 | 1.09 |  |  |  | 4.01 | 1.03 |  |  |  | — | — |  |  |  |

Note. SD = Standard Deviation; Skew = Skewness; Kurt = Kurtosis; Mdn = Median; Min = Minimum; Max = Maximum. Full sample excludes Occupational Wellness items (shown as —). Domain-Level Summary reports the mean of item means and the mean of item SDs per domain. Items rated on a 5-point Likert scale (1 = Never, 5 = Always). * Indicates reverse-scored items (Em3, Sp6); values reflect corrected reversed scores

**Appendix 2 Cross-Loadings in the ESEM of the Eight-Factor Well-Being Scale — Full Sample**

| **Item** | **Physical** | | **Emotional** | | **Home Environmental** | | **Social** | | **Spiritual** | | **Financial** | | **Mental** | | **Occupational** | |
| --- | --- | --- | --- | --- | --- | --- | --- | --- | --- | --- | --- | --- | --- | --- | --- | --- |
|  | **λ** | **SE** | **λ** | **SE** | **λ** | **SE** | **λ** | **SE** | **λ** | **SE** | **λ** | **SE** | **λ** | **SE** | **λ** | **SE** |
| **PHYSICAL** | | | | | | | | | | | | | | | | |
| PH2 | **0.250ᶜ** | *(0.036)* | 0.152ᶜ | *(0.044)* | 0.000 | *(0.042)* | -0.012 | *(0.051)* | 0.061 | *(0.050)* | 0.125ᵇ | *(0.039)* | 0.086ᵃ | *(0.038)* | — | — |
| PH5 | **0.224ᶜ** | *(0.050)* | 0.375ᶜ | *(0.054)* | -0.144ᵃ | *(0.067)* | 0.155ᵇ | *(0.052)* | 0.113ᵃ | *(0.049)* | 0.259ᶜ | *(0.039)* | -0.230ᶜ | *(0.042)* | — | — |
| PH6 | **0.330ᶜ** | *(0.037)* | 0.091 | *(0.052)* | -0.119ᵇ | *(0.040)* | 0.124ᵃ | *(0.051)* | 0.127ᵇ | *(0.049)* | 0.096ᵃ | *(0.040)* | -0.011 | *(0.037)* | — | — |
| PH7 | **0.831ᶜ** | *(0.023)* | -0.140ᶜ | *(0.038)* | 0.069 | *(0.047)* | 0.064 | *(0.047)* | -0.078 | *(0.045)* | -0.029 | *(0.032)* | -0.010 | *(0.032)* | — | — |
| PH8 | **0.805ᶜ** | *(0.022)* | -0.037 | *(0.034)* | 0.053 | *(0.033)* | 0.079ᵃ | *(0.040)* | -0.138ᵇ | *(0.050)* | 0.025 | *(0.029)* | 0.016 | *(0.025)* | — | — |
| PH9 | **0.812ᶜ** | *(0.023)* | 0.108ᵇ | *(0.036)* | -0.024 | *(0.031)* | -0.088ᵃ | *(0.037)* | 0.031 | *(0.043)* | 0.043 | *(0.030)* | -0.036 | *(0.027)* | — | — |
| PH10 | **0.766ᶜ** | *(0.027)* | 0.212ᶜ | *(0.037)* | -0.053 | *(0.028)* | -0.100ᵇ | *(0.038)* | 0.135ᶜ | *(0.040)* | 0.048 | *(0.031)* | -0.092ᶜ | *(0.026)* | — | — |
| **EMOTIONAL** | | | | | | | | | | | | | | | | |
| Em1 | 0.249ᶜ | *(0.031)* | **0.532ᶜ** | *(0.055)* | -0.220ᶜ | *(0.040)* | 0.059 | *(0.039)* | 0.099ᵃ | *(0.042)* | 0.005 | *(0.033)* | 0.147ᶜ | *(0.036)* | — | — |
| Em2 | 0.166ᶜ | *(0.029)* | **0.492ᶜ** | *(0.047)* | -0.144ᶜ | *(0.037)* | 0.153ᶜ | *(0.044)* | 0.073 | *(0.041)* | -0.020 | *(0.034)* | 0.166ᶜ | *(0.033)* | — | — |
| Em4 | 0.230ᶜ | *(0.029)* | **0.373ᶜ** | *(0.039)* | 0.014 | *(0.032)* | 0.225ᶜ | *(0.044)* | -0.052 | *(0.047)* | -0.111ᵇ | *(0.035)* | 0.184ᶜ | *(0.033)* | — | — |
| Em5 | 0.178ᶜ | *(0.027)* | **0.467ᶜ** | *(0.047)* | 0.150ᶜ | *(0.038)* | 0.162ᶜ | *(0.045)* | -0.196ᶜ | *(0.050)* | 0.002 | *(0.034)* | 0.218ᶜ | *(0.031)* | — | — |
| Em6 | 0.134ᶜ | *(0.029)* | **0.511ᶜ** | *(0.037)* | 0.092ᵃ | *(0.041)* | 0.100ᵃ | *(0.041)* | 0.070 | *(0.039)* | -0.088ᵇ | *(0.034)* | 0.178ᶜ | *(0.029)* | — | — |
| EM7 | 0.127ᶜ | *(0.028)* | **0.535ᶜ** | *(0.074)* | 0.342ᶜ | *(0.042)* | -0.077 | *(0.043)* | 0.197ᶜ | *(0.043)* | -0.097ᵇ | *(0.034)* | 0.113ᵇ | *(0.037)* | — | — |
| Em8 | 0.006 | *(0.030)* | **0.540ᶜ** | *(0.057)* | 0.261ᶜ | *(0.049)* | 0.046 | *(0.035)* | 0.215ᶜ | *(0.038)* | -0.048 | *(0.032)* | 0.123ᶜ | *(0.030)* | — | — |
| Em9 | -0.037 | *(0.028)* | **0.556ᶜ** | *(0.062)* | 0.293ᶜ | *(0.057)* | 0.065 | *(0.035)* | 0.090ᵃ | *(0.038)* | 0.122ᶜ | *(0.032)* | 0.074ᵃ | *(0.030)* | — | — |
| **HOME ENVIRONMENTAL** | | | | | | | | | | | | | | | | |
| EN1 | -0.007 | *(0.031)* | 0.441ᶜ | *(0.057)* | **0.304ᶜ** | *(0.066)* | 0.136ᶜ | *(0.039)* | 0.235ᶜ | *(0.034)* | 0.057 | *(0.032)* | 0.001 | *(0.031)* | — | — |
| En2 | 0.015 | *(0.028)* | 0.314ᶜ | *(0.048)* | **0.271ᶜ** | *(0.055)* | 0.181ᶜ | *(0.036)* | 0.127ᶜ | *(0.037)* | 0.160ᶜ | *(0.032)* | 0.081ᵇ | *(0.031)* | — | — |
| En3 | 0.125ᶜ | *(0.034)* | 0.230ᶜ | *(0.056)* | **0.274ᶜ** | *(0.072)* | 0.176ᵇ | *(0.055)* | -0.071 | *(0.054)* | 0.214ᶜ | *(0.041)* | -0.192ᶜ | *(0.038)* | — | — |
| En4 | -0.006 | *(0.032)* | 0.234ᶜ | *(0.039)* | **0.201ᵇ** | *(0.073)* | 0.398ᶜ | *(0.047)* | 0.058 | *(0.038)* | 0.232ᶜ | *(0.037)* | -0.231ᶜ | *(0.032)* | — | — |
| **SOCIAL** | | | | | | | | | | | | | | | | |
| Soci1 | -0.049 | *(0.032)* | 0.331ᶜ | *(0.037)* | 0.172ᵇ | *(0.065)* | **0.428ᶜ** | *(0.052)* | 0.062 | *(0.040)* | 0.216ᶜ | *(0.032)* | -0.126ᶜ | *(0.033)* | — | — |
| Soci2 | 0.025 | *(0.031)* | -0.013 | *(0.037)* | 0.107ᵇ | *(0.039)* | **0.652ᶜ** | *(0.049)* | -0.000 | *(0.045)* | 0.115ᶜ | *(0.033)* | -0.017 | *(0.032)* | — | — |
| Soci3 | 0.024 | *(0.029)* | -0.027 | *(0.040)* | -0.009 | *(0.031)* | **0.833ᶜ** | *(0.046)* | -0.063 | *(0.043)* | 0.008 | *(0.030)* | 0.034 | *(0.031)* | — | — |
| Soci4 | -0.013 | *(0.029)* | 0.000 | *(0.045)* | -0.076ᵃ | *(0.031)* | **0.850ᶜ** | *(0.042)* | 0.059 | *(0.037)* | -0.055 | *(0.032)* | 0.029 | *(0.033)* | — | — |
| Soci5 | 0.066ᵃ | *(0.031)* | 0.056 | *(0.036)* | -0.014 | *(0.031)* | **0.375ᶜ** | *(0.044)* | 0.248ᶜ | *(0.039)* | -0.006 | *(0.031)* | 0.215ᶜ | *(0.029)* | — | — |
| Soci6 | 0.126ᶜ | *(0.030)* | -0.151ᶜ | *(0.040)* | 0.142ᶜ | *(0.039)* | **0.504ᶜ** | *(0.047)* | 0.285ᶜ | *(0.039)* | -0.102ᵇ | *(0.032)* | 0.117ᶜ | *(0.030)* | — | — |
| Soci7 | 0.105ᶜ | *(0.029)* | -0.033 | *(0.038)* | 0.177ᶜ | *(0.034)* | **0.433ᶜ** | *(0.044)* | 0.232ᶜ | *(0.038)* | -0.089ᵇ | *(0.030)* | 0.239ᶜ | *(0.029)* | — | — |
| **SPIRITUAL** | | | | | | | | | | | | | | | | |
| Sp1 | 0.136ᶜ | *(0.034)* | -0.154ᵇ | *(0.050)* | 0.178ᶜ | *(0.035)* | 0.061 | *(0.044)* | **0.778ᶜ** | *(0.039)* | -0.002 | *(0.035)* | -0.013 | *(0.035)* | — | — |
| Sp2 | 0.016 | *(0.029)* | 0.036 | *(0.030)* | 0.069ᵃ | *(0.028)* | 0.055 | *(0.035)* | **0.874ᶜ** | *(0.028)* | -0.002 | *(0.029)* | 0.011 | *(0.025)* | — | — |
| Sp3 | -0.042 | *(0.029)* | 0.038 | *(0.027)* | -0.016 | *(0.028)* | 0.154ᶜ | *(0.038)* | **0.817ᶜ** | *(0.031)* | 0.025 | *(0.028)* | -0.015 | *(0.028)* | — | — |
| Sp4 | 0.020 | *(0.031)* | 0.013 | *(0.034)* | -0.000 | *(0.031)* | 0.037 | *(0.042)* | **0.855ᶜ** | *(0.032)* | 0.085ᵇ | *(0.029)* | -0.041 | *(0.029)* | — | — |
| Sp5 | -0.039 | *(0.036)* | 0.068 | *(0.042)* | -0.119ᶜ | *(0.036)* | 0.183ᶜ | *(0.043)* | **0.692ᶜ** | *(0.037)* | 0.105ᶜ | *(0.031)* | -0.008 | *(0.031)* | — | — |
| Sp7 | 0.047 | *(0.033)* | 0.052 | *(0.037)* | -0.015 | *(0.033)* | -0.023 | *(0.045)* | **0.464ᶜ** | *(0.044)* | 0.230ᶜ | *(0.034)* | 0.234ᶜ | *(0.032)* | — | — |
| **FINANCIAL** | | | | | | | | | | | | | | | | |
| Fin1 | 0.158ᶜ | *(0.027)* | -0.037 | *(0.032)* | 0.040 | *(0.031)* | -0.158ᶜ | *(0.038)* | 0.169ᶜ | *(0.047)* | **0.722ᶜ** | *(0.031)* | 0.025 | *(0.026)* | — | — |
| Fin2 | 0.073ᵇ | *(0.028)* | 0.010 | *(0.032)* | 0.035 | *(0.037)* | -0.069 | *(0.037)* | 0.136ᵇ | *(0.043)* | **0.764ᶜ** | *(0.031)* | -0.008 | *(0.028)* | — | — |
| Fin3 | 0.061ᵃ | *(0.026)* | -0.210ᶜ | *(0.031)* | 0.092ᶜ | *(0.027)* | 0.024 | *(0.036)* | 0.010 | *(0.039)* | **0.770ᶜ** | *(0.029)* | 0.170ᶜ | *(0.027)* | — | — |
| Fin4 | -0.020 | *(0.028)* | -0.067ᵃ | *(0.034)* | -0.039 | *(0.029)* | 0.202ᶜ | *(0.032)* | 0.067 | *(0.038)* | **0.619ᶜ** | *(0.030)* | 0.183ᶜ | *(0.027)* | — | — |
| Fin5 | 0.139ᶜ | *(0.028)* | -0.225ᶜ | *(0.040)* | 0.124ᵇ | *(0.038)* | 0.084ᵃ | *(0.040)* | -0.071 | *(0.046)* | **0.434ᶜ** | *(0.035)* | 0.419ᶜ | *(0.030)* | — | — |
| **MENTAL** | | | | | | | | | | | | | | | | |
| Ment1 | 0.086ᶜ | *(0.023)* | 0.062ᵃ | *(0.031)* | 0.085ᵇ | *(0.027)* | 0.037 | *(0.032)* | -0.030 | *(0.037)* | 0.154ᶜ | *(0.026)* | **0.670ᶜ** | *(0.025)* | — | — |
| Ment2 | 0.123ᶜ | *(0.027)* | 0.045 | *(0.034)* | 0.100ᶜ | *(0.029)* | 0.074 | *(0.041)* | -0.144ᶜ | *(0.043)* | 0.263ᶜ | *(0.029)* | **0.544ᶜ** | *(0.029)* | — | — |
| Ment3 | 0.072ᵇ | *(0.028)* | 0.063ᵃ | *(0.031)* | -0.032 | *(0.036)* | 0.101ᵇ | *(0.033)* | 0.006 | *(0.039)* | 0.059ᵃ | *(0.027)* | **0.623ᶜ** | *(0.024)* | — | — |
| Ment4 | -0.137ᶜ | *(0.031)* | 0.306ᶜ | *(0.033)* | -0.115ᶜ | *(0.030)* | 0.100ᵇ | *(0.036)* | 0.126ᶜ | *(0.038)* | 0.181ᶜ | *(0.025)* | **0.552ᶜ** | *(0.033)* | — | — |
| Ment5 | -0.076ᵇ | *(0.026)* | 0.278ᶜ | *(0.034)* | -0.133ᶜ | *(0.022)* | 0.093ᵇ | *(0.032)* | 0.149ᶜ | *(0.032)* | 0.103ᶜ | *(0.024)* | **0.634ᶜ** | *(0.029)* | — | — |
| Ment6 | -0.001 | *(0.028)* | 0.128ᶜ | *(0.030)* | 0.020 | *(0.024)* | 0.118ᶜ | *(0.036)* | 0.059 | *(0.038)* | 0.139ᶜ | *(0.029)* | **0.553ᶜ** | *(0.029)* | — | — |
| **OCCUPATIONAL** | | | | | | | | | | | | | | | | |
| Oc_E1 | — | — | — | — | — | — | — | — | — | — | — | — | — | — | — | — |
| Oc_E2 | — | — | — | — | — | — | — | — | — | — | — | — | — | — | — | — |
| Oc_E3 | — | — | — | — | — | — | — | — | — | — | — | — | — | — | — | — |
| Oc_E4 | — | — | — | — | — | — | — | — | — | — | — | — | — | — | — | — |
| Oc_E5 | — | — | — | — | — | — | — | — | — | — | — | — | — | — | — | — |
| Oc_E6 | — | — | — | — | — | — | — | — | — | — | — | — | — | — | — | — |
|  |  |  |  |  |  |  |  |  |  |  |  |  |  |  |  |  |
| *Note. ESEM = Exploratory Structural Equation Modeling. λ = standardized factor loading under WLSMV estimation, Theta parameterization, orthogonal target rotation. Primary (target) loadings are bolded and shaded. ᵃp < .05. ᵇp < .01. ᶜp < .001. — = not estimated in this group's model.* | | | | | | | | | | | | | | | | |

**Appendix 3 Cross-Loadings in the ESEM of the Eight-Factor Well-Being Scale — Employed**

| **Item** | **Physical** | | **Emotional** | | **Home Eniroment** | | **Social** | | **Spiritual** | | **Financial** | | **Mental** | | **Occupational** | |
| --- | --- | --- | --- | --- | --- | --- | --- | --- | --- | --- | --- | --- | --- | --- | --- | --- |
|  | **λ** | **SE** | **λ** | **SE** | **λ** | **SE** | **λ** | **SE** | **λ** | **SE** | **λ** | **SE** | **λ** | **SE** | **λ** | **SE** |
| **PHYSICAL** | | | | | | | | | | | | | | | | |
| PH2 | — | — | 0.174ᵇ | *(0.065)* | — | — | -0.163ᵃ | *(0.067)* | — | — | — | — | — | — | — | — |
| PH5 | **0.236ᶜ** | *(0.067)* | — | — | — | — | — | — | — | — | — | — | — | — | — | — |
| PH6 | **0.390ᶜ** | *(0.046)* | — | — | -0.137ᵃ | *(0.058)* | 0.200ᵇ | *(0.063)* | — | — | 0.113ᵃ | *(0.055)* | — | — | — | — |
| PH7 | **0.864ᶜ** | *(0.030)* | -0.124ᵃ | *(0.050)* | — | — | — | — | — | — | -0.094ᵃ | *(0.047)* | — | — | — | — |
| PH8 | **0.863ᶜ** | *(0.029)* | — | — | — | — | — | — | — | — | — | — | 0.086ᵃ | *(0.038)* | — | — |
| PH9 | **0.764ᶜ** | *(0.027)* | — | — | — | — | — | — | — | — | — | — | -0.083ᵃ | *(0.035)* | — | — |
| PH10 | **0.694ᶜ** | *(0.031)* | — | — | — | — | — | — | — | — | 0.083ᵃ | *(0.042)* | — | — | -0.102ᵇ | *(0.039)* |
| **EMOTIONAL** | | | | | | | | | | | | | | | | |
| Em1 | — | — | **0.626ᶜ** | *(0.048)* | — | — | — | — | — | — | — | — | 0.087ᵃ | *(0.043)* | — | — |
| Em2 | 0.137ᶜ | *(0.040)* | **0.518ᶜ** | *(0.048)* | — | — | 0.172ᶜ | *(0.052)* | — | — | — | — | 0.137ᶜ | *(0.041)* | — | — |
| Em4 | — | — | **0.542ᶜ** | *(0.052)* | — | — | 0.126ᵃ | *(0.055)* | — | — | — | — | — | — | — | — |
| Em5 | — | — | — | — | 0.086ᵃ | *(0.040)* | — | — | -0.261ᶜ | *(0.067)* | 0.112ᵃ | *(0.046)* | — | — | — | — |
| Em6 | — | — | **0.632ᶜ** | *(0.042)* | — | — | — | — | 0.156ᵇ | *(0.049)* | — | — | — | — | 0.121ᵇ | *(0.042)* |
| EM7 | — | — | **0.579ᶜ** | *(0.057)* | 0.389ᶜ | *(0.035)* | — | — | — | — | — | — | 0.081ᵃ | *(0.039)* | 0.086ᵃ | *(0.037)* |
| Em8 | — | — | **0.537ᶜ** | *(0.046)* | — | — | 0.110ᵇ | *(0.043)* | — | — | -0.102ᵃ | *(0.043)* | 0.087ᵃ | *(0.039)* | — | — |
| Em9 | — | — | **0.530ᶜ** | *(0.045)* | 0.305ᶜ | *(0.034)* | 0.134ᵇ | *(0.044)* | — | — | — | — | 0.078ᵃ | *(0.037)* | — | — |
| **HOME ENVIRONMENTAL** | | | | | | | | | | | | | | | | |
| EN1 | — | — | 0.385ᶜ | *(0.040)* | — | — | 0.096ᵃ | *(0.048)* | — | — | — | — | — | — | — | — |
| En2 | — | — | — | — | — | — | — | — | 0.132ᵃ | *(0.055)* | 0.111ᵃ | *(0.046)* | 0.103ᵇ | *(0.039)* | — | — |
| En3 | 0.106ᵃ | *(0.043)* | 0.153ᵇ | *(0.056)* | **0.436ᶜ** | *(0.052)* | — | — | — | — | 0.160ᵇ | *(0.052)* | -0.101ᵃ | *(0.045)* | — | — |
| En4 | — | — | 0.107ᵃ | *(0.042)* | — | — | — | — | — | — | — | — | — | — | — | — |
| **SOCIAL** | | | | | | | | | | | | | | | | |
| Soci1 | — | — | — | — | 0.331ᶜ | *(0.040)* | — | — | 0.113ᵇ | *(0.043)* | — | — | -0.102ᵃ | *(0.042)* | — | — |
| Soci2 | — | — | -0.111ᵃ | *(0.050)* | 0.143ᵇ | *(0.046)* | **0.657ᶜ** | *(0.059)* | — | — | 0.145ᵇ | *(0.049)* | — | — | — | — |
| Soci3 | — | — | — | — | — | — | **0.808ᶜ** | *(0.054)* | — | — | — | — | 0.083ᵃ | *(0.038)* | 0.075ᵃ | *(0.034)* |
| Soci4 | — | — | 0.111ᵇ | *(0.040)* | -0.094ᵃ | *(0.038)* | **0.815ᶜ** | *(0.048)* | — | — | -0.097ᵃ | *(0.038)* | — | — | — | — |
| Soci5 | 0.125ᵇ | *(0.044)* | — | — | — | — | — | — | — | — | — | — | — | — | — | — |
| Soci6 | 0.090ᵃ | *(0.043)* | — | — | 0.125ᵇ | *(0.043)* | — | — | — | — | -0.112ᵇ | *(0.042)* | — | — | 0.130ᶜ | *(0.039)* |
| Soci7 | 0.104ᵇ | *(0.039)* | — | — | 0.160ᶜ | *(0.042)* | — | — | 0.185ᶜ | *(0.054)* | — | — | — | — | — | — |
| **SPIRITUAL** | | | | | | | | | | | | | | | | |
| Sp1 | 0.141ᵇ | *(0.048)* | -0.173ᵇ | *(0.058)* | 0.138ᶜ | *(0.040)* | — | — | **0.725ᶜ** | *(0.057)* | — | — | — | — | 0.145ᵇ | *(0.050)* |
| Sp2 | — | — | — | — | 0.113ᵃ | *(0.044)* | — | — | **0.890ᶜ** | *(0.043)* | — | — | — | — | — | — |
| Sp3 | — | — | — | — | — | — | 0.188ᵇ | *(0.060)* | **0.761ᶜ** | *(0.049)* | 0.094ᵃ | *(0.045)* | — | — | — | — |
| Sp4 | — | — | — | — | — | — | — | — | **0.860ᶜ** | *(0.044)* | — | — | — | — | — | — |
| Sp5 | — | — | 0.122ᵇ | *(0.044)* | -0.086ᵃ | *(0.042)* | 0.193ᶜ | *(0.055)* | **0.663ᶜ** | *(0.051)* | 0.099ᵃ | *(0.047)* | — | — | — | — |
| Sp7 | — | — | — | — | — | — | — | — | — | — | — | — | — | — | — | — |
| **FINANCIAL** | | | | | | | | | | | | | | | | |
| Fin1 | — | — | — | — | — | — | -0.175ᶜ | *(0.047)* | — | — | **0.737ᶜ** | *(0.046)* | — | — | — | — |
| Fin2 | — | — | — | — | — | — | — | — | 0.129ᵃ | *(0.056)* | **0.720ᶜ** | *(0.047)* | — | — | — | — |
| Fin3 | — | — | -0.101ᵃ | *(0.048)* | 0.107ᵇ | *(0.037)* | — | — | — | — | **0.744ᶜ** | *(0.042)* | — | — | — | — |
| Fin4 | — | — | — | — | — | — | — | — | — | — | **0.598ᶜ** | *(0.045)* | — | — | — | — |
| Fin5 | — | — | — | — | 0.090ᵃ | *(0.042)* | 0.120ᵃ | *(0.047)* | -0.154ᵇ | *(0.059)* | **0.407ᶜ** | *(0.046)* | 0.383ᶜ | *(0.040)* | — | — |
| **MENTAL** | | | | | | | | | | | | | | | | |
| Ment1 | 0.122ᶜ | *(0.034)* | — | — | 0.108ᵇ | *(0.034)* | — | — | — | — | 0.154ᶜ | *(0.042)* | **0.613ᶜ** | *(0.035)* | 0.073ᵃ | *(0.035)* |
| Ment2 | 0.138ᶜ | *(0.038)* | — | — | — | — | — | — | -0.167ᵇ | *(0.054)* | — | — | **0.554ᶜ** | *(0.038)* | 0.086ᵃ | *(0.036)* |
| Ment3 | — | — | 0.092ᵃ | *(0.044)* | — | — | — | — | — | — | — | — | **0.587ᶜ** | *(0.037)* | — | — |
| Ment4 | — | — | 0.343ᶜ | *(0.039)* | — | — | 0.124ᵇ | *(0.048)* | 0.098ᵃ | *(0.048)* | 0.163ᶜ | *(0.042)* | **0.551ᶜ** | *(0.043)* | — | — |
| Ment5 | -0.111ᶜ | *(0.033)* | 0.294ᶜ | *(0.033)* | — | — | 0.087ᵃ | *(0.038)* | — | — | 0.093ᵃ | *(0.036)* | **0.616ᶜ** | *(0.039)* | — | — |
| Ment6 | — | — | — | — | — | — | — | — | 0.114ᵃ | *(0.047)* | 0.141ᵇ | *(0.044)* | **0.527ᶜ** | *(0.038)* | 0.109ᶜ | *(0.031)* |
| **OCCUPATIONAL** | | | | | | | | | | | | | | | | |
| Oc_E1 | — | — | — | — | — | — | — | — | 0.135ᵃ | *(0.054)* | 0.161ᶜ | *(0.047)* | — | — | **0.527ᶜ** | *(0.045)* |
| Oc_E2 | — | — | -0.089ᵃ | *(0.040)* | — | — | — | — | — | — | — | — | 0.071ᵃ | *(0.030)* | **0.929ᶜ** | *(0.021)* |
| Oc_E3 | — | — | — | — | — | — | — | — | -0.088ᵃ | *(0.041)* | — | — | — | — | **0.961ᶜ** | *(0.021)* |
| Oc_E4 | -0.104ᵇ | *(0.036)* | 0.124ᵃ | *(0.049)* | -0.125ᵇ | *(0.042)* | 0.120ᵇ | *(0.045)* | — | — | — | — | — | — | **0.811ᶜ** | *(0.032)* |
| Oc_E5 | 0.088ᵇ | *(0.033)* | -0.128ᵇ | *(0.048)* | — | — | — | — | — | — | — | — | — | — | **0.813ᶜ** | *(0.027)* |
| Oc_E6 | — | — | 0.117ᵃ | *(0.054)* | — | — | — | — | 0.135ᵇ | *(0.046)* | 0.095ᵃ | *(0.047)* | -0.134ᵇ | *(0.043)* | **0.614ᶜ** | *(0.044)* |
|  |  |  |  |  |  |  |  |  |  |  |  |  |  |  |  |  |
| *Note. ESEM = Exploratory Structural Equation Modeling. λ = standardized factor loading under WLSMV estimation, Theta parameterization, orthogonal target rotation. Primary (target) loadings are bolded and shaded. ᵃp < .05. ᵇp < .01. ᶜp < .001. — = not estimated in this group's model.* | | | | | | | | | | | | | | | | |

**Appendix 4 Cross-Loadings in the ESEM of the Eight-Factor Well-Being Scale — Non‑employed**

| **Item** | **Physical** | | **Emotional** | | **Home Environmental** | | **Social** | | **Spiritual** | | **Financial** | | **Mental** | | **Occupational** | |
| --- | --- | --- | --- | --- | --- | --- | --- | --- | --- | --- | --- | --- | --- | --- | --- | --- |
|  | **λ** | **SE** | **λ** | **SE** | **λ** | **SE** | **λ** | **SE** | **λ** | **SE** | **λ** | **SE** | **λ** | **SE** | **λ** | **SE** |
| **PHYSICAL** | | | | | | | | | | | | | | | | |
| PH2 | **0.194ᶜ** | *(0.049)* | 0.090 | *(0.058)* | 0.167ᶜ | *(0.047)* | 0.025 | *(0.056)* | 0.019 | *(0.057)* | 0.115ᵃ | *(0.056)* | 0.135ᵇ | *(0.049)* | 0.038 | *(0.062)* |
| PH5 | **0.240ᶜ** | *(0.068)* | 0.125 | *(0.066)* | 0.393ᶜ | *(0.049)* | -0.089 | *(0.066)* | 0.170ᵇ | *(0.056)* | 0.162ᵇ | *(0.060)* | -0.030 | *(0.053)* | 0.065 | *(0.064)* |
| PH6 | **0.293ᶜ** | *(0.056)* | 0.053 | *(0.061)* | 0.177ᶜ | *(0.049)* | 0.003 | *(0.061)* | 0.119ᵃ | *(0.055)* | 0.084 | *(0.055)* | 0.087 | *(0.050)* | -0.017 | *(0.066)* |
| PH7 | **0.786ᶜ** | *(0.035)* | -0.069 | *(0.052)* | -0.056 | *(0.046)* | 0.106 | *(0.056)* | -0.102 | *(0.054)* | 0.002 | *(0.049)* | 0.012 | *(0.045)* | -0.005 | *(0.050)* |
| PH8 | **0.776ᶜ** | *(0.034)* | -0.032 | *(0.048)* | -0.009 | *(0.046)* | 0.098ᵃ | *(0.042)* | -0.172ᵇ | *(0.062)* | 0.042 | *(0.044)* | -0.010 | *(0.035)* | 0.063 | *(0.044)* |
| PH9 | **0.815ᶜ** | *(0.034)* | 0.037 | *(0.045)* | -0.112ᵇ | *(0.043)* | -0.122ᵇ | *(0.042)* | 0.093 | *(0.051)* | -0.012 | *(0.044)* | -0.036 | *(0.038)* | 0.095ᵃ | *(0.046)* |
| PH10 | **0.809ᶜ** | *(0.040)* | 0.107ᵃ | *(0.048)* | -0.005 | *(0.041)* | -0.131ᵇ | *(0.040)* | 0.180ᶜ | *(0.052)* | 0.053 | *(0.049)* | -0.080ᵃ | *(0.038)* | -0.045 | *(0.050)* |
| **EMOTIONAL** | | | | | | | | | | | | | | | | |
| Em1 | 0.244ᶜ | *(0.043)* | **0.308ᶜ** | *(0.060)* | 0.218ᶜ | *(0.041)* | 0.026 | *(0.056)* | 0.099 | *(0.056)* | -0.004 | *(0.046)* | 0.222ᶜ | *(0.041)* | -0.039 | *(0.056)* |
| Em2 | 0.206ᶜ | *(0.041)* | **0.345ᶜ** | *(0.063)* | 0.170ᶜ | *(0.047)* | 0.029 | *(0.055)* | 0.065 | *(0.054)* | -0.060 | *(0.050)* | 0.209ᶜ | *(0.044)* | 0.073 | *(0.055)* |
| Em4 | 0.198ᶜ | *(0.045)* | **0.395ᶜ** | *(0.057)* | -0.066 | *(0.048)* | 0.227ᶜ | *(0.052)* | -0.047 | *(0.055)* | -0.060 | *(0.053)* | 0.087 | *(0.048)* | 0.051 | *(0.060)* |
| Em5 | 0.053 | *(0.040)* | **0.643ᶜ** | *(0.054)* | 0.033 | *(0.050)* | 0.213ᶜ | *(0.049)* | -0.253ᶜ | *(0.059)* | 0.022 | *(0.046)* | 0.126ᵇ | *(0.048)* | -0.004 | *(0.055)* |
| Em6 | 0.154ᶜ | *(0.039)* | **0.507ᶜ** | *(0.049)* | 0.094ᵃ | *(0.045)* | 0.213ᶜ | *(0.045)* | -0.109ᵃ | *(0.052)* | -0.065 | *(0.050)* | 0.187ᶜ | *(0.039)* | -0.013 | *(0.061)* |
| EM7 | 0.146ᶜ | *(0.041)* | **0.607ᶜ** | *(0.048)* | 0.031 | *(0.041)* | -0.006 | *(0.050)* | 0.177ᶜ | *(0.053)* | -0.049 | *(0.045)* | 0.034 | *(0.041)* | 0.041 | *(0.052)* |
| Em8 | -0.123ᵇ | *(0.041)* | **0.718ᶜ** | *(0.048)* | 0.012 | *(0.038)* | 0.053 | *(0.041)* | 0.139ᵇ | *(0.048)* | 0.113ᵇ | *(0.039)* | 0.076ᵃ | *(0.037)* | -0.003 | *(0.043)* |
| Em9 | -0.090ᵃ | *(0.044)* | **0.571ᶜ** | *(0.050)* | 0.152ᶜ | *(0.041)* | -0.013 | *(0.044)* | 0.094ᵃ | *(0.047)* | 0.130ᵇ | *(0.047)* | -0.024 | *(0.039)* | 0.197ᶜ | *(0.045)* |
| **HOME ENVIRONMENTAL** | | | | | | | | | | | | | | | | |
| EN1 | 0.098ᵃ | *(0.039)* | 0.347ᶜ | *(0.041)* | **0.389ᶜ** | *(0.046)* | 0.143ᶜ | *(0.042)* | 0.194ᶜ | *(0.040)* | -0.024 | *(0.045)* | -0.006 | *(0.040)* | 0.041 | *(0.048)* |
| En2 | 0.067 | *(0.040)* | 0.251ᶜ | *(0.044)* | **0.321ᶜ** | *(0.043)* | 0.185ᶜ | *(0.042)* | 0.074 | *(0.044)* | 0.127ᵇ | *(0.044)* | 0.061 | *(0.038)* | 0.077 | *(0.045)* |
| En3 | 0.164ᵇ | *(0.054)* | 0.193ᵇ | *(0.065)* | **0.209ᶜ** | *(0.061)* | 0.118 | *(0.065)* | -0.109 | *(0.066)* | 0.124ᵃ | *(0.061)* | -0.226ᶜ | *(0.051)* | 0.191ᵇ | *(0.067)* |
| En4 | 0.017 | *(0.047)* | 0.177ᶜ | *(0.052)* | **0.352ᶜ** | *(0.052)* | 0.204ᶜ | *(0.055)* | 0.112ᵃ | *(0.048)* | 0.098 | *(0.054)* | -0.176ᶜ | *(0.044)* | 0.179ᵇ | *(0.057)* |
| **SOCIAL** | | | | | | | | | | | | | | | | |
| Soci1 | -0.066 | *(0.051)* | 0.198ᶜ | *(0.052)* | 0.348ᶜ | *(0.042)* | **0.266ᶜ** | *(0.074)* | 0.068 | *(0.052)* | 0.110ᵃ | *(0.047)* | -0.077 | *(0.047)* | 0.240ᶜ | *(0.053)* |
| Soci2 | 0.031 | *(0.043)* | 0.024 | *(0.045)* | 0.212ᶜ | *(0.040)* | **0.510ᶜ** | *(0.060)* | 0.035 | *(0.049)* | 0.045 | *(0.044)* | 0.059 | *(0.041)* | 0.094ᵃ | *(0.047)* |
| Soci3 | 0.046 | *(0.043)* | 0.021 | *(0.047)* | 0.149ᶜ | *(0.041)* | **0.650ᶜ** | *(0.059)* | -0.017 | *(0.051)* | 0.060 | *(0.042)* | 0.015 | *(0.038)* | 0.043 | *(0.045)* |
| Soci4 | -0.066 | *(0.041)* | -0.027 | *(0.045)* | 0.070 | *(0.041)* | **0.714ᶜ** | *(0.056)* | 0.109ᵇ | *(0.042)* | 0.009 | *(0.047)* | 0.033 | *(0.040)* | 0.098ᵃ | *(0.045)* |
| Soci5 | 0.071 | *(0.045)* | 0.030 | *(0.049)* | 0.072 | *(0.044)* | **0.340ᶜ** | *(0.055)* | 0.276ᶜ | *(0.046)* | -0.015 | *(0.046)* | 0.246ᶜ | *(0.039)* | -0.027 | *(0.054)* |
| Soci6 | 0.052 | *(0.039)* | 0.150ᵇ | *(0.052)* | -0.209ᶜ | *(0.041)* | **0.554ᶜ** | *(0.057)* | 0.269ᶜ | *(0.047)* | 0.078 | *(0.047)* | -0.026 | *(0.038)* | -0.056 | *(0.046)* |
| Soci7 | 0.020 | *(0.039)* | 0.275ᶜ | *(0.043)* | -0.200ᶜ | *(0.039)* | **0.523ᶜ** | *(0.049)* | 0.205ᶜ | *(0.042)* | 0.021 | *(0.039)* | 0.097ᵇ | *(0.036)* | -0.018 | *(0.041)* |
| **SPIRITUAL** | | | | | | | | | | | | | | | | |
| Sp1 | 0.118ᵃ | *(0.049)* | 0.051 | *(0.061)* | -0.117ᵃ | *(0.055)* | 0.198ᶜ | *(0.047)* | **0.696ᶜ** | *(0.043)* | 0.040 | *(0.048)* | -0.131ᵇ | *(0.042)* | 0.088 | *(0.054)* |
| Sp2 | 0.027 | *(0.040)* | 0.028 | *(0.046)* | 0.061 | *(0.034)* | 0.162ᶜ | *(0.041)* | **0.744ᶜ** | *(0.038)* | 0.020 | *(0.043)* | 0.067ᵃ | *(0.033)* | 0.063 | *(0.047)* |
| Sp3 | -0.025 | *(0.042)* | 0.082 | *(0.053)* | 0.002 | *(0.045)* | 0.147ᶜ | *(0.038)* | **0.788ᶜ** | *(0.042)* | 0.017 | *(0.039)* | -0.016 | *(0.038)* | 0.072 | *(0.039)* |
| Sp4 | 0.092ᵃ | *(0.045)* | -0.080 | *(0.053)* | 0.129ᵇ | *(0.041)* | 0.078 | *(0.053)* | **0.737ᶜ** | *(0.043)* | 0.071 | *(0.045)* | 0.020 | *(0.041)* | 0.103 | *(0.055)* |
| Sp5 | 0.027 | *(0.052)* | -0.042 | *(0.065)* | 0.090ᵃ | *(0.044)* | 0.160ᵇ | *(0.052)* | **0.627ᶜ** | *(0.045)* | 0.114ᵃ | *(0.046)* | 0.076 | *(0.044)* | 0.059 | *(0.053)* |
| Sp7 | 0.049 | *(0.049)* | 0.124ᵃ | *(0.051)* | -0.077 | *(0.047)* | -0.005 | *(0.050)* | **0.411ᶜ** | *(0.053)* | 0.188ᶜ | *(0.048)* | 0.204ᶜ | *(0.047)* | 0.118ᵃ | *(0.049)* |
| **FINANCIAL** | | | | | | | | | | | | | | | | |
| Fin1 | 0.061 | *(0.037)* | 0.177ᶜ | *(0.045)* | -0.099ᵃ | *(0.047)* | -0.143ᵇ | *(0.047)* | 0.005 | *(0.057)* | **0.790ᶜ** | *(0.042)* | -0.107ᵇ | *(0.041)* | 0.049 | *(0.044)* |
| Fin2 | -0.004 | *(0.039)* | 0.158ᶜ | *(0.046)* | -0.049 | *(0.040)* | -0.097ᵃ | *(0.045)* | 0.050 | *(0.060)* | **0.900ᶜ** | *(0.042)* | -0.077 | *(0.043)* | -0.043 | *(0.044)* |
| Fin3 | 0.031 | *(0.035)* | -0.223ᶜ | *(0.042)* | 0.039 | *(0.033)* | 0.091ᵃ | *(0.044)* | 0.015 | *(0.048)* | **0.834ᶜ** | *(0.041)* | 0.113ᵇ | *(0.035)* | -0.035 | *(0.041)* |
| Fin4 | 0.020 | *(0.039)* | -0.161ᶜ | *(0.043)* | 0.132ᶜ | *(0.038)* | 0.173ᶜ | *(0.039)* | 0.062 | *(0.049)* | **0.665ᶜ** | *(0.045)* | 0.169ᶜ | *(0.036)* | -0.037 | *(0.044)* |
| Fin5 | 0.137ᶜ | *(0.041)* | -0.194ᶜ | *(0.049)* | -0.016 | *(0.042)* | 0.081 | *(0.049)* | -0.041 | *(0.058)* | **0.450ᶜ** | *(0.051)* | 0.348ᶜ | *(0.042)* | 0.069 | *(0.052)* |
| **MENTAL** | | | | | | | | | | | | | | | | |
| Ment1 | 0.073ᵃ | *(0.033)* | 0.134ᶜ | *(0.040)* | -0.129ᶜ | *(0.033)* | 0.088ᵃ | *(0.035)* | -0.027 | *(0.041)* | 0.162ᶜ | *(0.035)* | **0.610ᶜ** | *(0.034)* | 0.070ᵃ | *(0.034)* |
| Ment2 | 0.121ᵇ | *(0.039)* | 0.031 | *(0.044)* | -0.035 | *(0.040)* | 0.158ᶜ | *(0.047)* | -0.156ᵇ | *(0.055)* | 0.233ᶜ | *(0.041)* | **0.419ᶜ** | *(0.042)* | 0.125ᵃ | *(0.048)* |
| Ment3 | 0.077ᵃ | *(0.039)* | 0.101ᵃ | *(0.041)* | -0.169ᶜ | *(0.038)* | 0.057 | *(0.042)* | 0.020 | *(0.047)* | 0.076ᵃ | *(0.037)* | **0.589ᶜ** | *(0.037)* | 0.070 | *(0.046)* |
| Ment4 | -0.059 | *(0.041)* | 0.144ᶜ | *(0.044)* | 0.077ᵃ | *(0.031)* | 0.015 | *(0.037)* | 0.127ᵇ | *(0.048)* | 0.136ᶜ | *(0.029)* | **0.527ᶜ** | *(0.038)* | 0.140ᶜ | *(0.040)* |
| Ment5 | -0.000 | *(0.035)* | 0.151ᶜ | *(0.043)* | 0.075ᵃ | *(0.032)* | 0.018 | *(0.035)* | 0.098ᵃ | *(0.041)* | 0.063ᵃ | *(0.032)* | **0.654ᶜ** | *(0.035)* | 0.079ᵃ | *(0.037)* |
| Ment6 | -0.045 | *(0.039)* | 0.134ᵇ | *(0.044)* | -0.032 | *(0.037)* | 0.075 | *(0.039)* | 0.020 | *(0.047)* | 0.053 | *(0.041)* | **0.509ᶜ** | *(0.040)* | 0.242ᶜ | *(0.041)* |
| **OCCUPATIONAL** | | | | | | | | | | | | | | | | |
| Oc_E1 † | 0.004 | *(0.041)* | 0.071 | *(0.042)* | 0.132ᶜ | *(0.037)* | -0.171ᶜ | *(0.038)* | 0.247ᶜ | *(0.043)* | -0.054 | *(0.039)* | 0.218ᶜ | *(0.033)* | **0.610ᶜ** | *(0.049)* |
| Oc_E2 † | 0.042 | *(0.039)* | -0.015 | *(0.042)* | 0.170ᶜ | *(0.040)* | -0.003 | *(0.047)* | 0.146ᵇ | *(0.046)* | -0.086ᵃ | *(0.037)* | 0.164ᶜ | *(0.033)* | **0.645ᶜ** | *(0.051)* |
| Oc_E3 † | 0.025 | *(0.041)* | -0.058 | *(0.050)* | 0.043 | *(0.041)* | 0.086 | *(0.045)* | -0.063 | *(0.047)* | -0.009 | *(0.043)* | 0.113ᵇ | *(0.039)* | **0.700ᶜ** | *(0.052)* |
| Oc_E4 † | 0.032 | *(0.042)* | 0.055 | *(0.050)* | -0.100ᵃ | *(0.044)* | -0.003 | *(0.049)* | -0.149ᵃ | *(0.061)* | 0.051 | *(0.046)* | -0.041 | *(0.043)* | **0.812ᶜ** | *(0.060)* |
| Oc_E5 † | 0.055 | *(0.045)* | -0.108 | *(0.055)* | -0.177ᵇ | *(0.055)* | 0.110ᵃ | *(0.048)* | -0.060 | *(0.058)* | 0.032 | *(0.042)* | -0.066 | *(0.045)* | **0.817ᶜ** | *(0.050)* |
| Oc_E6 † | 0.005 | *(0.047)* | 0.032 | *(0.056)* | -0.022 | *(0.051)* | 0.002 | *(0.047)* | 0.219ᶜ | *(0.054)* | 0.065 | *(0.043)* | 0.010 | *(0.046)* | **0.519ᶜ** | *(0.049)* |
|  |  |  |  |  |  |  |  |  |  |  |  |  |  |  |  |  |
| *Note. ESEM = Exploratory Structural Equation Modeling. λ = standardized factor loading under WLSMV estimation, Theta parameterization, orthogonal target rotation. Primary (target) loadings are bolded and shaded. ᵃp < .05. ᵇp < .01. ᶜp < .001. — = not estimated in this group's model. † Non-Employed occupational items labeled Oc_NE1–Oc_NE6 (parallel to Oc_E1–Oc_E6).* | | | | | | | | | | | | | | | | |
